# Combining Strongly Orthogonal Geminals with Explicitly Correlated Corrections

**DOI:** 10.1021/acs.jctc.5c00264

**Published:** 2025-08-05

**Authors:** Zs. É. Mihálka, J. Noga

**Affiliations:** Department of Inorganic Chemistry, Faculty of Natural Sciences, 37864 Comenius University in Bratislava, Mlynská dolina, Ilkovičova 6, 842 15 Bratislava 4, Slovakia

## Abstract

Recent accounts of the good performance of the pair coupled cluster doubles (pCCD) method extended by an explicitly correlated (F12) model in describing essential correlation effects motivated us to further explore pair-function based methodologies in conjunction with the explicitly correlated framework. While pCCD can be reformulated as the antisymmetrized product of interacting two-electron fragments (geminals), driven by the special structure of the resulting reference function, we opt for employing strongly orthogonal geminals in the Ansatz. In this work, the antisymmetrized product of strongly orthogonal geminals wave function (SLG), which is capable of capturing static correlation in a computationally efficient manner, is corrected by an F12 addition via perturbation theory. The explicitly correlated terms of the wave function are internally contracted and are *entirely* responsible for describing the dynamic correlation missing from SLG, by virtue of incorporating all possible double excitations from the reference. A Slater-type function is used as a correlation factor, while amplitudes obtained from the variational optimization of the Hylleraas-functional as well as those fixed according to the *s*- and *p*-wave cusp conditions (SP-Ansatz) are explored. The presented SLG-F12 model is applied in order to investigate the splitting of the lowest singlet and triplet states in small biradicals and in other multireference systems.

## Graphical abstract

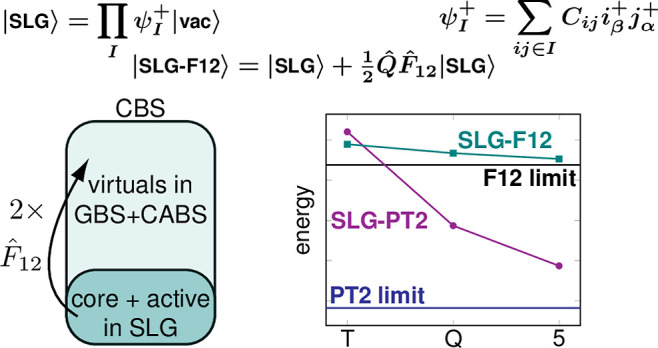

## 1 Introduction

Correct representation of the electronic structure requires a balanced treatment of static and dynamic effects. Most quantum chemical approaches follow a two-step protocol: obtain a reference wave function which captures the static part of electron correlation, then apply a correction which will provide the missing dynamic part. Strongly correlated systems, where the amount of static correlation is substantial, propose a challenge. Description of such systems calls for multireference (MR) approaches, most notably the complete active space self-consistent field (CASSCF) method.^1^ Dynamic correlation as well as MR variants of perturbation theory (PT), configuration interaction (CI) or coupled-cluster (CC) models are much more challenging both from a theoretical as well as a computational point of view. To reduce these difficulties, approaches combining MR wave functions with the single-reference (SR) formalism such as the state-selective MRCC theory^2–4^ or the family of tailored CC^5^ were proposed. Even with recent developments in this regard, such as the application of the density matrix renormalization group,^6–11^ quantum Monte Carlo approaches^12,13^ or the adaptive sampling configuration interaction method,^14^ the unfavorable scaling of CAS-based methods prompts the search for different models with lower computational demands.

Therefore, the alternative route that we also consider in this paper is to choose a more cost-effective reference instead of CASSCF. These include the unrestricted Hartree–Fock (UHF) wave function,^15^ models employing restricted active spaces^16–18^ or references with special structure such as the Generalized Valence Bond-Perfect Pairing (GVB-PP),^19^ Antisymmetrized Product of Strongly orthogonal Geminals (APSG),^20–22^ Strictly Localized Geminals (SLG),^23,24^ Strongly orthogonal Singlet-type Geminals (SSG)^25,26^ and the Antisymmetric Product of 1-reference orbital Geminals (AP1roG).^27^ A structurally compact reference can also potentially ensure the simplification of the dynamic correlation treatment built upon it, further reducing the computational complexity of the problem.

The feature linking UHF, GVB-PP, APSG, SLG, SSG and AP1roG is that they can all be expressed with the help of pair-functions (geminals), presenting themselves as different flavours of the same general theory. Geminal-based models can be considered as the straightforward generalization of the independent particle wave function,^28^ stepping from the one-particle view to pair-functions. This framework reflects the intuitive picture of electronic structure being represented by electron pairs (cf. Lewis structures). As intrapair correlation is incorporated at the reference level, pair-function based methods are capable of describing strongly correlated systems at a reasonable cost. Due to these features, geminal models are gaining momentum again: they are suitable for large scale computations,^29^ some variants form exactly solvable models,^30^ while others are applicable for attractive pair-interactions in all correlation regimes.^31–33^ For more details, we point to a recent review^34^ of geminal-based approaches. In order to incorporate dynamic electron correlation, various MR schemes can be adapted to the geminal reference, including PT,^35–40^ CC,^41,42^ CI^43^ and the extended random phase approximation.^44,45^

On the other hand, explicit incorporation of the interelectronic distance coordinate into the wave function is another tool for enhancing the quality of the dynamic correlation treatment.^46^ The emerging, explicitly correlated (denoted as R12 or F12) theory is also capable of improving the convergence of a given quantum chemical method with respect to the computational basis, by virtue of properly describing the electron–electron cusp.^47^ In most applications of the explicitly correlated framework, the R12/F12 correction is included *on top of* a classical method involving dynamic electron correlation. Accordingly, various MP2-F12 ^48–51^ CI-F12,^52,53^ CC-F12 ^54–58^ models have been developed, with the common feature that the F12 component of the Ansätze involves hypothetical excitations to the (infinite dimensional) orthogonal complement of the computational basis. In these approaches, all of the usual excitations are taken care of by the underlying conventional quantum chemical method. Varganov and Martínez^59^ considered a different route: in their F12-augmented multiconfigurational self-consistent field (MCSCF) method (denoted as G-MCSCF) dynamical correlation is treated entirely by an F12 factor, and the MCSCF wave function is optimized in the presence of this component.

In this work, we examine the possibility of combining the F12 framework with geminal-based reference functions. We are encouraged by the success of the relatively simple pair coupled-cluster doubles (pCCD) method extended by F12.^60^ It has been demonstrated that while pCCD is able to capture static correlation well, a significant amount of dynamic correlation is missing from the model,^34^ whereas inclusion of the explicitly correlated component on top of pCCD greatly improves the description of dynamic correlation.^60^ Since pCCD is equivalent to AP1roG,^61^ we are motivated to explore other geminal models in conjunction with the explicitly correlated scheme. Our reference is built as an antisymmetrized product of strongly orthogonal, formally spin-unrestricted geminals. In lieu of conventional dynamic correction, the explicitly correlated component includes excitations *within* the computational basis as well as excitations to its orthogonal complement, and is obtained by PT using a Dyall-like model Hamiltonian.^62^ By incorporating conventional double excitations in the F12 component instead of treating them separately, the explicitly correlated component shall have two tasks: to include dynamical correlation conventionally captured by a different correction method and to correctly describe the cusp of the wave function. Clearly, this parametrization will have much less variational degrees of freedom than an explicitly correlated, traditional dynamical treatment of the reference. Consequently, our aim with this work is to investigate the question: to what extent can the correlation be recovered by such a compact formulation of the Ansatz? Our approach of treating the conventional double excitations by F12 is not unique: Höfener and co-workers^63^ applied this idea successfully to the coupled cluster singles and perturbative doubles (CC2) theory. In their approach, singles were treated by the conventional CC formalism, while all double excitations, including conventional ones were included in the explicitly correlated component. The resulting CCS­(F12) model captures 80–95% of the CC2 correlation energy. In our approach, similarly to CCS­(F12) and to the work of Varganov and Martínez,^59^ the F12 component is intended to be entirely responsible for the description of the dynamic correlation, while the geminal reference shall account for the static part. In contrast with G-MCSCF, the pair-function based MR state will be optimized in advance, and the F12 correction is added *a posteriori* to the reference. In contrast with CCS­(F12) and pCCD-F12, we include the F12 component as a perturbation instead of treating it in a CC framework. We emphasize that in order to account for the effects usually treated by a separate dynamical correlation scheme, conventional excitations are also included in the explicitly correlated component.

The paper is organized as follows. In Section 2, we discuss the Ansatz in detail and derive the working equations. Computational details are given in Section 3, while numerical illustrations will be provided in Section 4 in the form of singlet–triplet gaps of small biradical systems.

## 2 Theory

### 2.1 Notation

We start from a multideterminantal reference function, which will be discussed in detail in Section 2.2. It follows that the given basis set (GBS) cannot be clearly divided into occupied and virtual orbitals. Therefore, the following notation is introduced and summarized in Table 1: *i*, *j*... denote *spatial* orbitals which are at least partially occupied in the reference, i.e., core and active orbitals. *a*, *b*, ... denote external spatial orbitals within the GBS. *p*, *q*, ... can stand for any orbital in the GBS, while κ, λ, ... label arbitrary orbitals from the complete basis set (CBS). Orbitals which are outside the GBS are denoted by α, β, ... (σ, α and β may also stand for spin, it is generally clear from the context whether the latter two denote spin or orbitals). External orbitals (either in GBS or in its complement) are denoted by *x*, *y*,.... The CBS (thus the GBS too) is assumed to be an orthonormal set. Any of the spatial orbitals can be appended by a spin index (σ = α or σ = β), and the corresponding creation and annihilation operators are μ_σ_^+^ and μ_σ_^–^, respectively. The reference is an antisymmetrized product of strongly orthogonal geminals, which will be indexed by capital latin letters (*I*, *J*, *K*, ...).

**Table 1:** Notation Used for Different Kinds of Orbitals

indices	subset	dimension
*p*, *q*, ...	GBS	*N*_basis_
*i*, *j*, ...	contributing to SLG	*N*_occ_ = *N*_core_ + *N*_act_
*a*, *b*, ...	GBS\{*i*, *j*, ...}	*N*_virt_
κ, λ, ...	CBS	
α, β, ...	CBS\GBS	
*x*, *y*, ...	{*a*, *b*, ...} ∪ {α, β, ...}	

### 2.2 Antisymmetrized Geminal Product Reference

The building blocks of our Ansatz are geminals, i.e., two-electron functions, which can be expressed as

1 $$\varphi_{I}^{+}=\sum_{ij}C_{ij}^{I}i_{\beta }^{+}j_{\alpha }^{+}$$

Strong orthogonality (SO) of geminals is enforced, which, according to Arai,^64^ is equivalent to geminals being expanded over pairwise disjoint subsets of the GBS. In the SO framework geminal subsets are thus nonoverlapping and the geminal index can be dropped from the coefficient matrix. Expansion of geminals is then restricted to a subset

2 $$\varphi_{I}^{+}=\sum_{ij}^{\left(I\right)}C_{ij}i_{\beta }^{+}j_{\alpha }^{+}$$

where the subset of the GBS corresponding to geminal *I* is also labeled by *I*. Dimension of the subspace spanned by the set *I* is denoted by *n*_*I*_. Geminals with *n*_*I*_ = 1 are referred to as core geminals. A core geminal is thus simply a doubly occupied orbital, which is also called core. Orbitals corresponding to geminals with *n*_*I*_ > 1 will be called active orbitals, while the corresponding two-electron units will be called correlated. Note, however, that a frozen core calculation does not necessarily mean that all core geminals are frozen; one-dimensional (core) geminals can take part in the correlation calculation. The reference is then obtained as

3 $$|SLG\rangle =\prod_{I=1}^{N/2}\varphi_{I}^{+}|vac\rangle$$

where *N* stands for the even number of electrons in the system. As is clear from eq 3, we only consider *M*_*S*_ = 0 systems. The acronym SLG is used throughout this work: it stands for strictly localized geminals. Localization in this case refers to localization in the Hilbert space due to the restricted summation.^23^ Symmetric part of ***C*** generates singlet geminals, while its antisymmetric part will generate a triplet component at the two-electron level. Consequently, the wave function in eq 3 is not spin pure in general.^25,40,65^

On top of being (strongly) orthogonal, geminals can be assumed to be normalized without loss of generality

$$\langle \varphi_{I}|\varphi_{J}\rangle =\delta_{IJ}\overset{\left(I\right)}{\underset{ij}{\sum }}{\left|C_{ij}\right|}^{2}=\delta_{IJ}$$ 4

which ensures normalization of |SLG⟩ as well. The parametrization given in eqs 2 and 3 leads to sparse reduced density matrices (RDMs), in particular, γ_*ij*_^σ^ = ⟨SLG|*i*_σ_^+^ *j*_σ_^–^|SLG⟩ is zero if *i* and *j* belong to different geminals, whereas $\Gamma_{ijlk}^{\sigma \sigma^{\prime }}=\left\langle SLG\left|i_{\sigma }^{+}j_{\sigma^{\prime }}^{+}k_{\sigma^{\prime }}^{-}l_{\sigma }^{-}\right|SLG\right\rangle$ is zero if there are more than two different geminals involved. For exact expressions, we point to ref 40. We also define γ_*ij*_ = γ_*ij*_^α^ + γ_*ij*_^β^.

The Ansatz of eq 3 can be variationally optimized on three different levels:

(i) according to geminal coefficients in ***C***,
(ii) by considering a unitary transformation of the GBS,
(iii) by optimizing the geminal subspace dimensions.

While the last option has also been explored,^25^ in most instances these numbers are typically given a priori and are kept fixed during the calculations. As mentioned, several methods including UHF, GVB-PP, APSG and SSG belong to the family of strongly orthogonal geminals’ framework. While AP1roG deals with interacting (nonorthogonal) pair-functions, it can still be considered as a generalization of the singlet-coupled methods such as GVB-PP and APSG by relaxing some of the restrictions on the geminal coefficient matrices, leading to the parametrization of AP1roG.

Optimization with respect to ***C*** is carried out by setting the expectation value of the Hamiltonian stationary.^66^ The resulting equations can be recast as two-electron pseudoeigenvalue problems

5 $${Ĥ}_{I}\varphi_{I,\xi }=E_{I,\xi }\varphi_{I,\xi },,\left(I=1,...,N/2\right)$$

where ${Ĥ}_{I}$ is the effective geminal Hamiltonian and *E*_*I*_ are called the geminal energies, ξ labeling geminal states (in practice, indices are given so that the geminal entering SLG has index ξ = 0, and if it is clear from the context, may omit ξ from the notation). Equation 5 are not independent, since ${Ĥ}_{I}$ includes intergeminal effects in a mean-field-like manner

6 $${Ĥ}_{I}=\sum_{ij}^{\left(I\right)}\sum_{\sigma }h_{ij}^{eff,\sigma }i_{\sigma }^{+}j_{\sigma }^{-}+\frac{1}{2}\sum_{ijkl}^{\left(I\right)}\sum_{\sigma ,\sigma^{\prime }}g_{ij}^{kl}i_{\sigma }^{+}j_{\sigma^{\prime }}^{+}l_{\sigma^{\prime }}^{-}k_{\sigma }^{-}$$

where σ, σ′ ∈ {α, β} and

7 $$h_{ij}^{eff,\sigma }=h_{ij}+\sum_{J\neq I_{i},I_{j}}\sum_{mn}^{\left(J\right)}\left(g_{im}^{jn}\gamma_{mn}-g_{im}^{nj}\gamma_{mn}^{\sigma }\right)$$

In eq 7 *I*_*i*_ refers to the subset that orbital *i* belongs to, *h*_*ij*_ are the one-electron integrals, and *g*_*pq*_^*rs*^ = [*pq*|*rs*] are the two-electron repulsion integrals (in 1212 convention). Perturbation theory (PT) based dynamic correlation treatments heavily rely on the existence of the effective operators of eq 6, and we will also utilize them in this work.

Orbital optimization can be carried out in an MCSCF-like manner,^67,68^ by a direct minimization process^25^ or other numerical algorithms.^69^ In order to maximize the simplicity of our model, we avoid orbital optimization altogether and use a predefined set of spatial orbitals as our GBS. Based on the suggestion by Pulay^70–72^ for defining active spaces, we follow the idea proposed by Földvári et al. in ref 40 and use the natural orbitals of the UHF wave function (UNOs) as the computational MO basis.

The resulting model is termed UNO-SLG. Geminal subspaces are defined based on the pairing scheme of UNOs, yielding either one-dimensional geminals (*n*_*I*_ = 1) built from a single doubly occupied UNO, or two-dimensional geminals (*n*_*I*_ = 2) expanded over two paired UNOs which have occupation numbers (in the UHF wave function) that add up to two. For details on the pairing of UNOs, we refer to works by Löwdin^73^ and Mayer,^74,75^ while its utilization in the context of SLG is elaborated in ref 40. This choice resembles the GVB-PP wave function with UNOs instead of optimized orbitals, however, in contrast with GVB-PP, which is restricted to singlet geminals, we allow triplet components at the two-electron level. This parametrization of the SLG Ansatz is one of the most compact choices but is capable of capturing essential static effects.

#### 2.2.1 Perturbing SLG

Since optimal geminals constituting SLG obey the eigenvalue equations given in eq 5, there is a straightforward choice for a zero-order Hamiltonian in a PT framework. Namely, if

8 $${Ĥ}^{\left(0\right)}:=\sum_{I=1}^{N/2}{Ĥ}_{I}+{Ĥ}_{ext}$$

where in its second-quantized form, ${Ĥ}_{ext}$ only involves orbitals from {*a*, *b*, ...}, then the zero-order equation

9 $${Ĥ}^{\left(0\right)}|SLG\rangle =\left(\sum_{I=1}^{N/2}E_{I}\right)|SLG\rangle$$

is automatically fulfilled. In previous works,^36,40^ two choices were considered for ${Ĥ}_{ext}$

10a $${Ĥ}_{V1}:=\sum_{ab}\sum_{\sigma }h_{ab}^{eff,\sigma }a_{\sigma }^{+}b_{\sigma }^{-}$$

10b $${Ĥ}_{V2}:=\sum_{ab}\sum_{\sigma }h_{ab}^{eff,\sigma }a_{\sigma }^{+}b_{\sigma }^{-}+\frac{1}{2}\sum_{abcd}\sum_{\sigma ,\sigma^{\prime }}g_{ab}^{cd}a_{\sigma }^{+}b_{\sigma^{\prime }}^{+}d_{\sigma^{\prime }}^{-}c_{\sigma }^{-}$$

The matrix elements *h*_*pq*_^eff,σ^ are obtained by generalizing eq 7

11a $$h_{pq}^{eff,\sigma }=h_{pq}+\sum_{\begin{array}{l} J=1\\ J\neq I_{p},I_{q} \end{array}}^{N/2}\sum_{mn}^{\left(J\right)}\left(g_{pm}^{qn}\gamma_{mn}-g_{pm}^{nq}\gamma_{mn}^{\sigma }\right)$$

11b $$h_{\kappa \lambda }^{\sigma }=h_{\kappa \lambda }+\sum_{J=1}^{N/2}\sum_{mn}^{\left(J\right)}\left(g_{\kappa m}^{\lambda n}\gamma_{mn}-g_{\kappa m}^{n\lambda }\gamma_{mn}^{\sigma }\right)$$

Equation 11b is also defined for later use. *h*_κλ_^σ^ only differs from *h*_κλ_^eff,σ^ if at least one of the orbital indices belongs to the set {*i*, *j*, ...}.

A crucial property of the zero-order operator of eq 8 (with any of the choices in eqs 10a and 10b) is that it preserves the number of electrons and the *z*-component of spin within each geminal subspace as well as the virtual subspace.

### 2.3 Explicitly Correlated Correction to SLG

Improved description of dynamic correlation as well as enhancement of basis set convergence are the main roles of the explicitly correlated extension of a model. Today, almost all methodologies model short-range effects by an exponentially parametrized F12 correlation factor (Slater-type geminals^76^ or a combination of Gaussian-type geminals^77^).[Fn fn1] MR techniques are commonly applied to describe systems with a significant amount of static correlation. Many of the standard MR approaches have their F12 counterpart: explicit correlation has been incorporated into MRPT,^78–80^ MRCI^52,53^ as well as MRCC.^81–83^ A general *a posteriori* Ansatz has been proposed by Torheyden and Valeev,^84^ dubbed [2]_*R*12_ as well as its spin-adapted variant^85^ SF-[2]_*R*12_, which can be used with any multiconfigurational reference wave function provided that the RDMs are available. In its simplest form, it can be applied to a CASSCF reference with the additional conventional MRCI correction.

Our approach, which will be generally called SLG-F12, is inspired by [2]_*R*12_ (and SF-[2]_*R*12_) in the sense that SLG-F12 is an *a posteriori* perturbative F12 correction to a multireference function, however, it differs from [2]_*R*12_ in three main points:

(i) there is no conventional dynamic correlation added to the reference,
(ii) consequently, standard double excitations are included in the F12 component,
(iii) SLG-F12 uses a Dyall-type partitioning, including 2-body terms in the zero-order Hamiltonian.

We note that Kong and Valeev have also developed an explicitly correlated correction to CASSCF,^86^ [2]_*S*_, which accounts for its basis set incompleteness. On one hand, our approach is similar to [2]_*S*_ since they are both perturbative, moreover, one variant of [2]_*S*_ also uses a Dyall-like partitioning. On the other hand, the first order interacting spaces are entirely different: we use double excitations to describe the correlation, while for CASSCF + [2]_*S*_ the effect of doubles (and most of the dynamic correlation) are intended to be incorporated by some additional MR method (e.g., MRCI).

#### 2.3.1 SLG-F12

The reference wave function is corrected by an explicitly correlated term as

$$|\Psi \rangle =|SLG\rangle +\frac{1}{2}\mathrm{Q̂}\mathrm{F̂}|SLG\rangle$$ 12

where F̂ generates a contracted explicitly correlated function from the multideterminantal SLG reference

13 $$\mathrm{F̂}=\sum_{kl}\sum_{ij}\sum_{\kappa \lambda }\sum_{\sigma \sigma^{\prime }}t_{kl}^{ij}\left(\sigma ,\sigma^{\prime }\right)F_{\kappa \lambda }^{kl}\kappa_{\sigma }^{+}\lambda_{\sigma^{\prime }}^{+}j_{\sigma^{\prime }}^{-}i_{\sigma }^{-}$$

while the projector Q̂ ensures orthogonality between SLG and the F12 correction. The role of Q̂ is to exclude those terms from the correction which were already accounted for in the reference. The integrals in eq 13 are the two-electron integrals over the correlation factor *f*_12_ = *f*(*r*_12_)­

14 $$F_{\kappa \lambda }^{\mu \nu }=\left\langle \kappa \lambda \left|f_{12}\right|\mu \nu \right\rangle =\iint \kappa^{*}\left(r_{1}\right)\lambda^{*}\left(r_{2}\right)f_{12}\mu \left(r_{1}\right)\nu \left(r_{2}\right)dr_{1}dr_{2}$$

and *t*_*kl*_^*ij*^(σ,σ^′^) are the explicitly correlated amplitudes which parametrize the correction. For brevity, we also introduce the notation

15 $$F_{\sigma ,\sigma^{\prime }}^{kl+}:=\sum_{\kappa ,\lambda }F_{\kappa \lambda }^{kl}\kappa_{\sigma }^{+}\lambda_{\sigma^{\prime }}^{+}$$

We treat $\frac{1}{2}\mathrm{Q̂}\mathrm{F̂}|SLG\rangle$ as a perturbative correction to SLG

16 $$|\Psi^{\left(1\right)}\rangle =\frac{1}{2}\mathrm{Q̂}\sum_{kl}\sum_{ij}\sum_{\sigma \sigma^{\prime }}t_{kl}^{ij}\left(\sigma ,\sigma^{\prime }\right)F_{\sigma ,\sigma^{\prime }}^{kl+}j_{\sigma^{\prime }}^{-}i_{\sigma }^{-}|SLG\rangle$$

and use the extended definition of ${Ĥ}_{ext}$ in the partitioning of the Hamiltonian. Indices *k*, *l*, *i*, *j* run over the occupied orbitals (i.e., core + active set). The zero-order operator has the form of eq 8 with possible choices for ${Ĥ}_{ext}$ as

17a $${Ĥ}_{ext1}:=\sum_{xy}\;\sum_{\sigma }h_{xy}^{\sigma }x_{\sigma }^{+}y_{\sigma }^{-}$$

$${Ĥ}_{ext2}:=\underset{xy}{\sum }\underset{\sigma }{\sum }h_{xy}^{\sigma }x_{\sigma }^{+}y_{\sigma }^{-}+\frac{1}{2}\underset{xyzw}{\sum }\underset{\sigma ,\sigma^{\prime }}{\sum }g_{xy}^{zw}x_{\sigma }^{+}y_{\sigma^{\prime }}^{+}w_{\sigma^{\prime }}^{-}z_{\sigma }^{-}$$ 17b

While ${Ĥ}_{ext1}$ contains only one-body terms, ${Ĥ}_{ext2}$ is a two-particle operator. The energy correction brought forth by |Ψ^(1)^⟩ is calculated from the Hylleraas-functional, i.e.

18 $$E^{\left(2\right)}=\left\langle \Psi^{\left(1\right)}\left|{Ĥ}^{\left(0\right)}-E^{\left(0\right)}\right|\Psi^{\left(1\right)}\right\rangle +\left\langle SLG\left|Ĥ\right|\Psi^{\left(1\right)}\right\rangle +\left\langle \Psi^{\left(1\right)}\left|Ĥ\right|SLG\right\rangle$$

F12 amplitudes are either obtained by setting $E^{\left(2\right)}$ stationary (variationally optimized amplitudes) or by fixing them according to the SP-Ansatz of Ten-no.^87^ The cusp conditions are automatically fulfilled if the SP-Ansatz is employed, and for single-reference methods it has several other advantages: it is unitary invariant, and there is no need to solve F12 amplitude equations, making it a computationally inexpensive choice. These properties ensured that the fixed amplitude method is prevalent in standard F12 models. After considering certain symmetries, eq 16 is reformulated as

19 $$|\Psi^{\left(1\right)}\rangle =\mathrm{Q̂}\sum_{kl}\sum_{ij}t_{kl}^{ij}\left(\alpha ,\beta \right)F_{\alpha ,\beta }^{kl+}j_{\beta }^{-}i_{\alpha }^{-}|SLG\rangle +2\mathrm{Q̂}\sum_{k<l}\sum_{i>j}\sum_{\sigma }t_{kl}^{ij}\left(\sigma ,\sigma \right)F_{\sigma ,\sigma }^{kl+}j_{\sigma }^{-}i_{\sigma }^{-}|SLG\rangle$$

#### 2.3.2 Transformed Amplitudes

Strong orthogonality allows the explicitly correlated amplitudes *t*_*kl*_^*ij*^ to be categorized based on which geminals indices *i* and *j* belong to. Namely, if *i*, *j* ∈ *I*, then the α–α (β–β) amplitudes are irrelevant, since trying to remove two electrons with the same spin from a single geminal will annihilate the SLG wave function. The internally contracted form of the explicitly correlated correction will generate terms which are linearly dependent. To avoid any ambiguities and to simplify the relevant expressions, it is convenient to introduce a combination of the original F12 amplitudes, generated by the geminal coefficients. They are defined as

20 $$T_{kl}^{I}=-\sum_{ij}^{\left(I\right)}C_{ji}t_{kl}^{ij}\left(\alpha ,\beta \right)$$

for any geminal I, and for any pair of geminals *I* ≠ *J* we write

21 $$T_{kl}^{mn}\left(\alpha ,\beta \right):=\sum_{i}^{\left(I\right)}\sum_{j}^{\left(J\right)}t_{kl}^{ij}\left(\alpha ,\beta \right)C_{mi}C_{jn}\;\forall m\in I,n\in J$$

Finally, for *I* > *J* we define

22 Tklmn(α,α):=−∑i(I)∑j(J)tklij(α,α)CmiCnj∀m∈I,n∈JTklmn(β,β):=−∑i(I)∑j(J)tklij(β,β)CimCjn∀m∈I,n∈J

We will refer to the above quantities as transformed amplitudes. The short-hand notation $|\Phi_{\mathrm{I̅}}\rangle =\prod_{J\neq I}\varphi_{J}^{+}|vac\rangle$ and $|\Phi_{\mathrm{I̅}\mathrm{J̅}}^{m_{\sigma }n_{\sigma^{\prime }}}\rangle =m_{\sigma }^{+}n_{\sigma^{\prime }}^{+}\prod_{K\neq I,J}\varphi_{K}^{+}|vac\rangle$ is also introduced. With the help of these, eq 19 can be rewritten as

$$\begin{array}{l} |\Psi^{\left(1\right)}\rangle =\underset{I}{\sum }\underset{kl}{\sum }T_{kl}^{I}\left(\alpha ,\beta \right)\mathrm{Q̂}F_{\alpha ,\beta }^{kl+}|\Phi_{\mathrm{I̅}}\rangle +\underset{I\neq J}{\sum }\overset{\left(I\right)}{\underset{m}{\sum }}\overset{\left(J\right)}{\underset{n}{\sum }}\underset{kl}{\sum }T_{kl}^{mn}\left(\alpha ,\beta \right)\mathrm{Q̂}F_{\alpha ,\beta }^{kl+}|\Phi_{\overset{®}{IJ}}^{m_{\beta }n_{\alpha }}\rangle \\ +2\underset{\sigma }{\sum }\underset{I>J}{\sum }\overset{\left(I\right)}{\underset{m}{\sum }}\overset{\left(J\right)}{\underset{n}{\sum }}\underset{k<l}{\sum }T_{kl}^{mn}\left(\sigma ,\sigma \right)\mathrm{Q̂}F_{\sigma ,\sigma }^{kl+}|\Phi_{\overset{®}{IJ}}^{m_{\mathrm{\sigma ̅}}n_{\mathrm{\sigma ̅}}}\rangle \end{array}$$ 23

with α̅ = β and β̅ = α.

In particular, if the SP-Ansatz^87^ is applied to the *t*_*kl*_^*ij*^ amplitudes, i.e., $t_{kl}^{ij}\left(\alpha ,\beta \right)=\frac{3}{8}\delta_{ik}\delta_{lj}+\frac{1}{8}\delta_{il}\delta_{jk}$ and $t_{kl}^{ij}\left(\sigma ,\sigma \right)=\frac{1}{8}\delta_{ik}\delta_{jl}-\frac{1}{8}\delta_{il}\delta_{jk}$ then the corresponding transformed quantities will be simply

TklISP=−δIkIδIlI(38Clk+18Ckl)TklmnSP(α,β)=38δIkIδIlJCmkCln+18δIlIδIkJCmlCknTklmnSP(α,α)=−18δIkIδIlJCmkCnl+18δIlIδIkJCmlCnkTklmnSP(β,β)=−18δIkIδIlJCkmCln+18δIlIδIkJClmCkn 24

#### 2.3.3 Projection Operators

The projector Q̂ keeps |Ψ^(1)^⟩ and |SLG⟩ orthogonal (cf. intermediate normalization), but its exact form requires some discussion. We will formulate it in the *N*-electron CI space, but if applicable, it will also be noted if its action can be equivalently expressed as a restricted summation on κ, λ in $F_{\sigma ,\sigma^{\prime }}^{kl+}$ . To better understand the distinct projection cases, let us write the summation in eq 15 as

25 $$\sum_{\kappa ,\lambda }=\sum_{x,y}+\sum_{x,m}+\sum_{m,x}+\sum_{m,n}$$

The first term on the rhs is responsible for purely external (double) excitations, the second and third terms correspond to semi-internal excitations, which can have components that are singly excited. The last term on the rhs generates fully internal type excitations, having both singles and doubles contributions. Since from the point of view from the SLG wave function, there is no distinction between the external orbitals *a* or α, we use their union for the F12 correction, i.e., *x*. The distinct excitation types span different subsets of the *N*-electron CI space, and we define the corresponding projectors accordingly: ${\mathrm{P̂}}_{SLG}=|SLG\rangle \langle SLG|$ ; ${\mathrm{P̂}}_{gem}$ projects onto the space spanned by $\left\{\prod_{I=1}^{N/2}\varphi_{I,\xi_{I}}^{+}|vac\rangle \right\}$ , i.e., it is the projector of the model space; ${\mathrm{P̂}}_{mn,ij}$ is the projector of the space spanned by $\left\{m_{\sigma }^{+}n_{\sigma^{\prime }}^{+}j_{\sigma^{\prime }}^{-}i_{\sigma }^{-}|SLG\rangle \right\}$ ; ${\mathrm{P̂}}_{xm,ij}$ projects onto the space spanned by $\left\{x_{\sigma }^{+}m_{\sigma^{\prime }}^{+}j_{\sigma^{\prime }}^{-}i_{\sigma }^{-}|SLG\rangle \right\}$ ; ${\mathrm{P̂}}_{m,i}$ the projector of the space generated by {*m*_σ_^+^ *i*_σ_^–^|SLG⟩}; and ${\mathrm{P̂}}_{x,i}$ projects onto the span of {*x*_σ_^+^ *i*_σ_^–^|SLG⟩}.

To obtain the desired orthogonality ⟨SLG|Ψ^(1)^⟩ = 0, it would be sufficient to choose $\mathrm{Q̂}=1-{\mathrm{P̂}}_{SLG}$ . In this case, individual configurations in the model space are not excluded, just the specific combination which results in SLG. Unlike in the case of CASSCF, these configurations can give nonzero contribution to the first-order wave function correction. To examine their effect, we also tested the projector $\mathrm{Q̂}=1-{\mathrm{P̂}}_{gem}$ , since the states constructed from the eigenfunctions of the geminal operators actually span the model space. Such a choice implies that we omit certain configurations resulting from the last term on the rhs of eq 25. An even further restriction is obtained if we use $\mathrm{Q̂}=1-{\mathrm{P̂}}_{mn,ij}$ , i.e., we omit all internal excitations, which is equivalent to dropping *∑*_*m*,*n*_ from eq 15. We also consider keeping only fully external excitations by using $\mathrm{Q̂}=1-{\mathrm{P̂}}_{xm,ij}-{\mathrm{P̂}}_{mn,ij}$ .

The question of singles remains to be discussed. The internally contracted semi-internal and fully internal excitations can have contributions which are not purely two-electron effects, i.e., that can actually be described by single excitations. The importance of singles in the F12 model has been emphasized both at the single-refs 56,88–90 and multirefs^86,91^ level. It can account for the basis set incompleteness error of the reference as well as help with the one-particle basis set relaxation in the case of a smaller GBS. In all of these instances, singles are treated separately from doubles, therefore we also project out the singles components of the semi-internal and internal excitations in this work. While the proper inclusion of singles (i.e., independently from the doubles) would probably be beneficial to our framework for the same reasons as it is so for CASSCF, we refrain from doing so. This is done in order to keep the model as simple as possible, and we plan to examine the effect of the singles correction in a future study. Finally, omission of singles will decouple the equations, reducing the computational cost of the model.

To summarize, we will consider the following projectors in the SLG-F12 Ansatz of eq 23:

(i)
$\mathrm{Q̂}=1-{\mathrm{P̂}}_{xm,ij}-{\mathrm{P̂}}_{mn,ij}$ —only fully external doubles are kept,
(ii)
$\mathrm{Q̂}=1-{\mathrm{P̂}}_{mn,ij}-{\mathrm{P̂}}_{x,i}$ —fully external and semi-internal doubles are present,
(iii)
$\mathrm{Q̂}=1-{\mathrm{P̂}}_{gem}-{\mathrm{P̂}}_{m,i}-{\mathrm{P̂}}_{x,i}+{\mathrm{P̂}}_{m,i}{\mathrm{P̂}}_{gem}$ —fully external, semi-internal and fully internal doubles are kept, with some specific internal configurations omitted,
(iv)
$\mathrm{Q̂}=1-{\mathrm{P̂}}_{m,i}-{\mathrm{P̂}}_{x,i}$ —all fully external, semi-internal and fully internal doubles are kept.

The resulting variant of the current SLG-F12 theory will be referred to as E, E + SI, E + SI + I′ and E + SI + I, respectively. We emphasize that the unconventional nature of the present projection operators is due to the fact that there is no other dynamic correlation treatment included in the Ansatz, nor is it assumed to be available. Therefore, to get a meaningful correction, the F12 terms need to at least partially include those configurations which are usually accounted for by other methods (CI, PT, CC) and thus are missing from the conventional F12 Ansätze.

#### 2.3.4 Working Equations

The structure of the zero-order operator in eq 8 (with any of the choices in eqs 17a and 17b) ensures that pure double excitations and the corresponding amplitudes will only interact through ${Ĥ}^{\left(0\right)}$ if the geminals involved in the excitation are the same. Therefore, if singles are not present, the Hylleraas-functional can be evaluated and optimized blockwise according to geminal pairs. To be precise, in this case

⟨ΦI′®|Fα,βk′l′−Q̂(Ĥ(0)−E(0))Q̂Fα,βkl+|ΦI̅⟩=δI′IHk′l′,klI⟨ΦI′®|Fα,βk′l′−Q̂(Ĥ(0)−E(0))Q̂Fα,βkl+|ΦIJ®mβnα⟩=0⟨ΦI′®|Fα,βk′l′−Q̂(Ĥ(0)−E(0))Q̂Fσ,σkl+|ΦIJ®mσ¯nσ¯⟩=0⟨ΦI′J′®mβ′nα′|Fα,βk′l′−Q̂(Ĥ(0)−E(0))Q̂Fα,βkl+|ΦIJ®mβnα⟩=δI′IδJ′JHk′l′m′n′,klmnIJ+δI′JδJ′IHk′l′m′n′,klmnIJ×2⟨ΦI′J′®mβ′nα′|Fα,βk′l′−Q̂(Ĥ(0)−E(0))Q̂Fσ,σkl+|ΦIJ®mσ¯nσ¯⟩=δI′IδJ′JHk′l′m′n′,klmnIJσ+δI′JδJ′IHk′l′m′n′,klmnIJσ×4⟨ΦI′J′®m′σ′®n′σ′®|Fσ′,σ′k′l′−Q̂(Ĥ(0)−E(0))Q̂Fσ,σkl+|ΦIJ®mσ̅nσ̅⟩=δI′IδJ′JHk′l′m′n′,klmnIJσ′σ 26

Since fully external, semi-internal and fully internal excitations do not interact via ${Ĥ}^{\left(0\right)}$ due to having different number of electrons in the external space, matrix elements H appearing in eq 26 will have these three distinct contributions. Evaluation of the matrix elements requires formal summation over the CBS. Resulting quantities are closely related to the conventional X , P , B and V matrices^49,57^ (stemming from fully external doubles) and Y matrices (obtained from the semi-internal doubles). To avoid many-electron integrals, we used the resolution of identity (RI) approach of Kutzelnigg^92^ as well as the complementary auxiliary basis set (CABS) approach of Valeev,^93^ followed by standard approximation^48^ (SA). Approximation C^51^ was employed to evaluate the B -analogous quantities. Detailed expressions are given in Appendix A. In this setup, variational optimization of the Hylleraas-functional will lead to the following equations for each pair of geminals *I* > *J*

27 $$\left(\begin{array}{llll} H^{IJ} & H^{JI\times } & H^{IJ\alpha } & H^{IJ\beta }\\ H^{IJ\times } & H^{JI} & H^{JI\alpha \times } & H^{JI\beta \times }\\ {\left(H^{IJ\alpha }\right)}^{†} & {\left(H^{JI\alpha \times }\right)}^{†} & H^{IJ\alpha \alpha } & H^{IJ\alpha \beta }\\ {\left(H^{IJ\beta }\right)}^{†} & {\left(H^{JI\beta \times }\right)}^{†} & H^{IJ\beta \alpha } & H^{IJ\beta \beta } \end{array}\right)\cdot \left(\begin{array}{l} T^{IJ\alpha \beta }\\ T^{JI\alpha \beta }\\ T^{IJ\alpha \alpha }\\ T^{IJ\beta \beta } \end{array}\right)+\left(\begin{array}{l} b^{IJ\alpha \beta }\\ b^{JI\alpha \beta }\\ b^{IJ\alpha \alpha }\\ b^{IJ\beta \beta } \end{array}\right)=0$$

where amplitudes *T*_*kl*_^*mn*^(α,β) with *m* ∈ *I*, *n* ∈ *J* are collected into the array ***T*** ^*IJ*αβ^, with analogous definitions for ***T*** ^*IJ*αα^ and ***T*** ^*IJ*ββ^. Moreover, elements of the arrays ***b*** ^*IJ*αβ^ and ***b*** ^*IJ*σσ^ are given by

28 $$\begin{array}{l} b_{klmn}^{\alpha ,\beta }=\left\langle \Phi_{\overset{®}{IJ}}^{m_{\beta }n_{\alpha }}\left|F_{\alpha ,\beta }^{kl-}Ĥ\right|SLG\right\rangle \\ b_{klmn}^{\sigma ,\sigma }=\left\langle \Phi_{\overset{®}{IJ}}^{m_{\mathrm{\sigma ̅}}n_{\mathrm{\sigma ̅}}}\left|F_{\sigma ,\sigma }^{kl-}Ĥ\right|SLG\right\rangle \end{array}$$

Optimized amplitudes *T*_*kl*_^*I*^, collected in ***T*** ^*I*^ will obey

29 $$H^{I}T^{I}+b^{I}=0$$

for each individual geminal *I* with $b_{kl}^{I}=\left\langle \Phi_{\mathrm{I̅}}\left|F_{\alpha ,\beta }^{kl-}Ĥ\right|SLG\right\rangle$ . We emphasize that if {*I* > *J*} is different from {*I*′ > *J*′}, then the corresponding eqs with the form of eq 27 can be solved separately for the two index pairs.

If fully internal excitations are completely removed $\left(\mathrm{Q̂}=1-{\mathrm{P̂}}_{mn,ij}-{\mathrm{P̂}}_{x,i}\right)$ , then $H^{IJ\times }$ , $H^{JI\times }$ , $H^{IJ\alpha \beta }$ and $H^{IJ\beta \alpha }$ vanish. Furthermore, if only doubly external states are kept $\left(\mathrm{Q̂}=1-{\mathrm{P̂}}_{mn,ij}-{\mathrm{P̂}}_{xm,ij}\right)$ , then $H^{IJ\sigma }$ and $H^{JI\sigma \times }$ will also become zero, leading to a block-diagonal (thus further decoupled) form of eq 27.

Finally, we mention the choice to use the SP-fixed amplitudes^87^ instead of the variationally optimized ones. In that case, the amplitudes of eq 24 are substituted into $E^{\left(2\right)}\left(T\right)=T^{†}HT+T^{†}b+b^{†}T$ to get the energy correction.

Computational cost of the SLG-F12 schemes is remarkably low. Preparation of all necessary integrals and intermediate matrices is at most a single $O\left(N_{basis}^{4}N_{occ}^{2}\right)$ step, the latter being facilitated by the relatively simple expressions obtained via SA (cf. Appendix A). The SLG reference can be obtained in $O\left(N_{gem}m^{4}\right)$ steps, where *N*_gem_ = *N*/2 is the number of geminals, while *m* = max_*I*_(*n*_*I*_), i.e., the maximal geminal subspace dimension (in UNO-SLG, *m* = 2). The explicitly correlated correction is derived from the Hylleraas-functional. Since it can be evaluated blockwise, optimization requires us to solve $O\left(N_{gem}^{2}\right)$ linear systems of equations, each of which has a dimension of at most $O\left(m^{2}N_{occ}^{2}\times m^{2}N_{occ}^{2}\right)$ . In comparison, the most demanding part of the conventional SLG-PT2 corresponds to the solution of $O\left(N_{gem}^{2}\right)$ linear systems, each having $O\left(m^{2}N_{virt}^{2}\times m^{2}N_{virt}^{2}\right)$ size. As often *N*_virt_ ≫ *N*_occ_ holds, SLG-F12 is in general considerably less expensive than SLG-PT2.

## 3 Computational Details

In each of the following applications, the GBS was chosen to be the set of UNOs. The geminal subspaces were defined based on the pairing scheme of the NOs of the *M*_*S*_ = 0 UHF solution. While the Ansatz in eq 3 is in general spin-contaminated, we avoided using geminal product wave functions which have spin impurities as it can hinder the performance of the dynamic correlation treatment built upon them. In cases where the lowest energy SLG function was spin-mixed, acquiring a spin pure SLG solution was achieved by starting the iterative solution process of eq 5 from a spin pure initial guess. Plugging geminals with correct spin symmetry into eq 6 results in ${Ĥ}_{I}$ commuting with ${Ŝ}^{2}$ and ensures that roots of eq 5 are also spin pure. When targeting ground or lowest lying excited states, out of the spin pure roots the pair function with lowest geminal energy possessing the correct spin symmetry was chosen. While the spin pure solution obtained this way may not be a fully optimal SLG state (i.e., there may be a spin-mixed solution lower in energy), the geminals constituting the spin pure SLG solutions do fulfill eq 5.

The F12 integrals and intermediates were obtained by using the correlation factor $f_{12}=-\frac{1}{\gamma }\exp\left(-\gamma r_{12}\right)$ with γ = 1.0 *a*_0_^–1^. Since they were evaluated within SA,^48^ auxiliary basis sets were not defined. We tested two different partitionings for the SLG-F12 scheme, namely, whether we keep the two electron part of ${Ĥ}_{ext}$ or not (cf. eqs 17a and 17b). In the latter case, the contributions from the two-electron repulsion integrals are dropped from the matrix elements (e.g., matrix P in A.4b, A.5, and A.8) and the corresponding terms in the *G* matrices listed in Table 10).

The SLG reference was obtained from the Budapest version of the Mungauss program,^94^ while the pair-function based F12 correction was implemented in the DirCCR12-OS program.^95^

## 4 Results

Pilot applications of the SLG-F12 scheme concern the singlet–triplet gaps of small molecules with biradical singlet state (O_2_, NH and CH_2_), and the ozone molecule, a challenging system for electronic structure methods. The pair-function based reference is an adequate tool for targeting such systems.^40,96,97^ Stretching of bonds as well as interaction curves are not examined as the UHF solution underlying the SLG framework might not be consistent for such cases. Calculations were performed using the cc-pVXZ basis sets, with, unless otherwise stated, frozen core post-SLG methods. A general observation in all our tests was that the DZ results are usually notably different from the data obtained with larger basis sets. Total energies may be completely off, and energetic order of states can be incorrect in the DZ basis. This can be attributed to the small size of the basis, since the assumptions of the SA (which is used in our explicitly correlated treatment) will not be properly met for cc-pVDZ. Another issue encountered was the different structure of the geminal reference in DZ vs larger basis sets. The alpha-beta splitting of the UHF solution is less prominent than in larger bases, as such, there may be less correlated geminals in the SLG reference in DZ basis than in TZ/QZ etc. The different structure makes comparison of the results difficult. Hence, we decided to omit the DZ data altogether, and focus on TZ/QZ/5Z values. Also, for brevity, in the following tables we do not show the results with the variant E + SI of the SLG-F12, which provided the most inaccurate results for the singlet–triplet gaps. Those results are available in the Supporting Information.

### 4.1 O_2_

Using the experimental geometries,^98^ the adiabatic energy splitting of the ^3^Σ ground and ^1^Δ excited state of the oxygen molecule are assessed by the SLG-F12 methods in cc-pVXZ^99^ basis sets. At the level of the geminal reference, all geminals apart from the cores are correlated (i.e., six two-dimensional units), while in a post-SLG correlation calculation, either all geminals are participating, or one can perform a frozen core calculation by omitting the two lowest-lying core orbitals (and corresponding geminals) from the PT2 and F12 treatments. We have tested both options, in the spirit of ref 88. For the triplet geometry, lowest energy SLG solutions are spin-contaminated, with $\left\langle {Ŝ}^{2}\right\rangle$ ranging from 1.66 to 1.63. To avoid spin-contamination, we sought for spin pure triplet solutions which are ca. 3 mE_h_ above their spin mixed counterparts. In the triplet SLG solution, which was used in the subsequent F12 calculations, all but one geminals are singlet, while the bonding geminal built from two π-type UNOs (with occupation numbers 1.0) is a pure triplet. Table 2 presents energetic data for the triplet ground state with all electrons or valence electrons correlated, respectively, for both external partitionings considered. Fully correlated calculations in cc-pCVXZ^100^ basis sets are also included. We compare total energies to standard SLG-PT2,^40^ while singlet–triplet splittings are compared to SLG-PT2, results from spin-flip based methodologies^101^ and experimental data.

**Table 2:** Total Energy (in E_h_) of the ^3^Σ Ground State of the Oxygen Molecule in cc-pVXZ and cc-pCVXZ Basis Sets, Obtained by SLG-Based Methods^a^

	core and valence electrons correlated					
	${Ĥ}_{ext2}$			${Ĥ}_{ext1}$		
	cc-pVTZ	cc-pVQZ	cc-pV5Z	cc-pVTZ	cc-pVQZ	cc-pV5Z
SLG	–149.65671	–149.66794	–149.67078	–149.65671	–149.66794	–149.67078
PT2	–150.11395	–150.19754	–150.22913	–150.13860	–150.23035	–150.26626
E	–150.02450	–149.98400	–149.98131	–150.06966	–150.02564	–150.02157
E + SI + I′	–150.22650	–150.19462	–150.19357	–150.27881	–150.24324	–150.24102
E + SI + I	–150.24442	–150.21389	–150.21279	–150.29795	–150.26273	–150.26047
E(SP)	–149.87023	–149.86839	–149.86642	–149.93384	–149.92356	–149.92031
E + SI + I′(SP)	–150.02631	–150.02519	–150.02200	–150.08990	–150.08036	–150.07589
E + SI + I(SP)	–150.03637	–150.03506	–150.03169	–150.09996	–150.09023	–150.08559
Valence Electrons Correlated						
PT2	–150.08864	–150.13991	–150.15909	–150.11263	–150.17058	–150.19340
E	–149.86484	–149.87369	–149.87777	–149.89806	–149.90671	–149.91103
E + SI + I′	–150.05934	–150.07479	–150.08010	–150.09971	–150.11510	–150.12080
E + SI + I	–150.07823	–150.09396	–150.09927	–150.11883	–150.13452	–150.14021
E(SP)	–149.84877	–149.86088	–149.86530	–149.88814	–149.89867	–149.90303
E + SI + I′(SP)	–150.00897	–150.02396	–150.02831	–150.04834	–150.06175	–150.06604
E + SI + I(SP)	–150.01904	–150.03383	–150.03800	–150.05841	–150.07162	–150.07574

	all electrons correlated					
	${Ĥ}_{ext2}$			${Ĥ}_{ext1}$		
	cc-pCVTZ	cc-pCVQZ	cc-pCV5Z	cc-pCVTZ	cc-pCVQZ	cc-pCV5Z
SLG	–149.65734	–149.66820	–149.67082	–149.65734	–149.66820	–149.67082
E	–149.97235	–149.97608	–149.97985	–150.01393	–150.01643	–150.01994
E + SI + I′	–150.18153	–150.18849	–150.19264	–150.22939	–150.23528	–150.23976
E + SI + I	–150.20055	–150.20782	–150.21189	–150.24858	–150.25485	–150.25924
E (SP)	–149.87638	–149.87723	–149.88021	–149.93031	–149.93020	–149.93327
E + SI + I′ (SP)	–150.02154	–150.02792	–150.03143	–150.07546	–150.08089	–150.08449
E + SI + I (SP)	–150.03162	–150.03779	–150.04113	–150.08555	–150.09076	–150.09419

a For cc-pVXZ results of fully correlated and frozen core post-SLG calculations are also reported. PT2 is the SLG-PT2 method of ref 40 methods E, E + SI + I′ and E + SI + I are variants of the SLG-F12 scheme, see text for their precise meaning. SP: the SP-Ansatz is used.

Three common tendencies can be observed for cases when all electrons or only valence electrons are correlated, which stand for both external partitionings. One, for a given basis total energies decrease as more configurations are included in the Ansatz (i.e., going from E to E + SI + I as less and less excitations are projected out as per Section 2.3.3). Since the variational flexibility (the number of explicitly correlated amplitudes) is the same for all SLG-F12 variants, this trend might be attributed to the similar structure of the E/SI/I contributions to $E^{\left(2\right)}$ . However, there is no rigorous proof and while we have not encountered any, there may be cases where the tendency changes. Two, total energies by the geminal-based F12 schemes using the SP-Ansatz are always above their variationally optimized variants, which was to be expected. Three, energies in a given basis by a given method are lower if the 1-particle external zero-order Hamiltonian is used in the partitioning, compared to the 2-particle ${Ĥ}_{ext2}$ case. This can be easily explained as the Hylleraas-functional for the two partitionings differs in a term that is derived from the two-electron repulsion integrals: expressions with P are present for the 2-body case but are absent from the 1-body external operator. The presence of P will increase the energy.

A distinct difference between fully correlated and frozen core calculations in cc-pVXZ is the convergence pattern of the total energies as the basis is expanded: for the former, absolute energies form an increasing sequence for all SLG-F12 schemes, whereas for frozen core calculations, total energies converge from above. While converging from below is not unheard of,^81^ this is relatively uncommon among conventional electron correlation methods. The unusual trend for the fully correlated calculations is, however, not present if the core–valence polarized cc-pCVXZ basis is employed, with total energies converging from above. Although this is not necessarily so (cf. fully correlated MP2-R12 results in ref 88), the mismatch between the basis and the chosen method can cause unbalanced result, as cc-pVXZ does not treat core and valence orbitals on the same footing. However, the method of ref 88 uses conventional orbital expansions as well as extended sets to approximate the RI. Therefore, the unconventional convergence pattern of the fully correlated cc-pVXZ results is caused by the combination of different factors: the lack of conventional orbital expansions, the different RI approximation and the basis mismatch. We thank one of our reviewers for indirectly prompting us to investigate this issue. Based on Table 2 any of the SLG-F12 approaches converges faster than SLG-PT2. Their limit does not coincide with that of SLG-PT2, however, this is not considered to be an error of these models. Since the SLG-F12 framework describes the dynamic correlation with less parameters and in a more contracted form, we cannot expect that it will yield the same basis set limit as SLG-PT2. Still, for the fully correlated case, the more involved Ansätze (those including semi-internal and fully internal excitations, i.e., E + SI + I′ and E + SI + I) with variational F12 amplitudes perform similarly to conventional PT. For frozen core calculations the variant incorporating the largest amount of correlation (E + SI + I) will recover 85% (87%) of the SLG-PT2 correlation energy in cc-pV5Z basis, for the external partitioning ${Ĥ}_{ext2}$ $\left({Ĥ}_{ext1}\right)$ . Another contrasting feature of all or just valence electrons being correlated is the difference between variational SLG-F12 values and the corresponding SP-fixed results. For fully correlated calculations, energies by the SP-Ansatz are 100–200 mE_h_ above the variational counterparts, whereas the difference becomes 10–60 mE_h_ for the frozen core case. We also note that for the ${Ĥ}_{ext2}$ partitioning and variants E + SI + I′ and E + SI (cf. Table S1 in Supporting Information) using the SP-Ansatz, frozen core results are below their fully correlated counterparts. This further confirms the findings in refs 102 and 103 that the SP-Ansatz is not adequate for core effects if the basis set is not matched.

Distinguishing between the methods becomes easier when the adiabatic excitation energies are examined. The dissimilar patterns followed by fully correlated and frozen core total energies are not reflected in the singlet–triplet splittings, collected in Table 3 for the frozen core calculations and Table S2 in the Supporting Information for the fully correlated case. Difference between corresponding entries in Tables 3 and S1 in the Supporting Information is 0.02 eV or less. SLG by itself overestimates the energy difference by ca. 0.3 eV, compared to the experimental gap. Addition of standard PT2 considerably reduces the error of the excitation energy, with results falling close to SF-OD. Energy differences by SLG-based F12 methods converge faster than those by conventional PT. However, performance of the explicitly correlated schemes varies among the different variants. Inclusion of semi-internal excitations is only meaningful if fully internal configurations are also present in the Ansatz (E + SI in Table S2 in Supporting Information vs E + SI + I(′)). This is true for both the variational and the SP-fixed amplitudes. Variants including semi-internal and internal excitations in the F12 component (E + SI + I′ and E + SI + I) yield the most accurate gaps, with the partitioning using ${Ĥ}_{ext1}$ being slightly better. These values are comparable to both the SLG-PT2 ones as well as to either SF-CIS­(D) or the more accurate SF-OD. Somewhat surprisingly, the less sophisticated SLG-F12 scheme using only external configurations (E) provides acceptable gaps. Based on the clearly less accurate total energies (compared to the more intricate variants), this result is likely due to error cancellation.

**Table 3:** Adiabatic Singlet–Triplet Gap (*E*(^1^Δ) – *E*(^3^Σ), in eV) of O_2_ in cc-pVXZ Basis Sets

	${Ĥ}_{ext2}$			${Ĥ}_{ext1}$		
	cc-pVTZ	cc-pVQZ	cc-pV5Z	cc-pVTZ	cc-pVQZ	cc-pV5Z
SLG	1.318	1.315	1.314	1.318	1.315	1.314
PT2	1.098	1.067	1.055	1.079	1.044	1.028
E	1.188	1.195	1.194	1.159	1.169	1.169
E + SI + I′	1.099	1.100	1.099	1.066	1.072	1.072
E + SI + I	1.077	1.077	1.075	1.043	1.046	1.045
E(SP)	1.198	1.205	1.205	1.160	1.170	1.170
E + SI + I′(SP)	1.226	1.233	1.233	1.188	1.197	1.199
E + SI + I(SP)	1.224	1.230	1.231	1.185	1.195	1.196
SF-CIS^a^	1.445	1.447				
SF-CIS(D)^a^	1.089	1.067				
SF-OD^a^	1.076	1.061				
expt.^b^	0.980					

a Reference 101.

b Reference 98.

### 4.2 NH

The triplet ground state and the lowest singlet excited state of the NH molecule is assessed next. Adiabatic gaps are calculated in cc-pVXZ basis sets, with experimental geometries taken from ref 98. Once again, we compare the quality of the SLG-F12 gaps to conventional geminal PT2, spin-flip approaches and experiment. Splitting of alpha and beta orbitals in the *M*_*S*_ = 0 UHF solution allows us to form 3 correlated geminals in the SLG reference. For the triplet geometry, the lowest in energy SLG solution is a spin pure triplet and acts as our reference.

Absolute energies by the SLG-based methods are collected in Table 4. The general tendencies are the same as for O_2_: energies by SLG-F12 variants decrease in the order of E, E + SI + I′, E + SI + I. Frozen core energies by the geminal-based F12 models form a decreasing sequence, converging significantly faster than conventional SLG-PT2. This is especially true for the Ansätze using the SP-fixed amplitudes. None of the variants tend to the limit of SLG-PT2, but that is not unexpected, as they approach the issue of dynamic correlation differently. Energies by the most inclusive Ansatz (E + SI + I, only components proportional to |SLG⟩ are projected out from the F12 correction) are closest to data by PT2, providing 87–89% of the SLG-PT2 correlation energy in cc-pV5Z basis. The models operating with a 1-body operator for the external space $\left({Ĥ}_{ext1}\right)$ provide lower values compared to the partitioning using the 2-particle ${Ĥ}_{ext2}$ . Energies with the SP-fixed approaches are naturally above those obtained by the matching variationally optimized models, recovering 80% of the SLG-PT2 correlation energy.

**Table 4:** Total Energy (in E_h_) of the ^3^Σ Ground State of NH in cc-pVXZ Basis Sets, Obtained by SLG-Based Methods

	${Ĥ}_{ext2}$			${Ĥ}_{ext1}$		
	cc-pVTZ	cc-pVQZ	cc-pV5Z	cc-pVTZ	cc-pVQZ	cc-pV5Z
SLG	–54.97348	–54.97703	–54.97795	–54.97348	–54.97703	–54.97795
PT2	–55.10171	–55.11490	–55.11933	–55.11722	–55.13278	–55.13841
E	–55.06035	–55.06298	–55.06403	–55.07767	–55.08014	–55.08120
E + SI + I′	–55.08714	–55.09090	–55.09203	–55.10624	–55.10980	–55.11098
E + SI + I	–55.09644	–55.10041	–55.10155	–55.11568	–55.11945	–55.12064
E (SP)	–55.05599	–55.05927	–55.06039	–55.07200	–55.07469	–55.07577
E + SI + I′ (SP)	–55.08179	–55.08567	–55.08678	–55.09780	–55.10109	–55.10217
E + SI + I (SP)	–55.08743	–55.09122	–55.09227	–55.10344	–55.10664	–55.10765

Interesting tendencies can be observed about the adiabatic gaps presented in Table 5. UNO-SLG by itself overestimates the singlet–triplet gap by about 0.2 eV. Adding the regular PT2 correction shifts the gap in the correct direction, however, the shift is too large and as a result the gap is underestimated by almost as much. In this regard, results by the explicitly correlated schemes with optimized amplitudes are not much different: they are of similar quality than those by SLG-PT2. Excitation energies by the schemes involving semi-internal and internal excitations within the F12 correction (E + SI + I′ and E + SI + I) are off by ca. −0.2 eV, an error of similar magnitude than the error of the spin-flip (SF) approach SF-CIS, and much less accurate than the more sophisticated SF methods. A striking exception is the simplest variant which includes only doubly external excitations in the F12 component (variant E). Even though absolute energies are not particularly accurate, it yields remarkably good singlet–triplet gaps, even more so with the ${Ĥ}_{ext2}$ partitioning. The models employing the SP-Ansatz provide stable gap sequences which are surprisingly close to experimental data, especially for the ${Ĥ}_{ext2}$ partitioning.

**Table 5:** Adiabatic Gap (*E*(^1^Δ) – *E*(^3^Σ) in eV) of NH in cc-pVXZ Basis Sets, Obtained by SLG-Based Methods

	${Ĥ}_{ext2}$			${Ĥ}_{ext1}$		
	cc-pVTZ	cc-pVQZ	cc-pV5Z	cc-pVTZ	cc-pVQZ	cc-pV5Z
SLG	1.771	1.751	1.743	1.771	1.751	1.743
PT2	1.482	1.404	1.376	1.448	1.362	1.329
E	1.563	1.555	1.551	1.512	1.508	1.504
E + SI + I′	1.378	1.339	1.329	1.342	1.307	1.297
E + SI + I	1.366	1.326	1.315	1.329	1.292	1.282
E (SP)	1.570	1.561	1.556	1.505	1.500	1.497
E + SI + I′ (SP)	1.597	1.578	1.571	1.533	1.518	1.512
E + SI + I (SP)	1.596	1.577	1.570	1.531	1.512	1.510
SF-CIS^a^	1.778	1.764				
SF-CIS(D)^a^	1.661	1.611				
SF-OD^a^	1.658	1.605				
expt.^b^	1.558					

a Reference 101.

b Reference 98.

### 4.3 CH_2_

As a relatively small biradical species, methylene (CH_2_) is a standard test system for multireference methods as well as experimental studies. It has a triplet ^3^ *B*_1_ ground state, while the lowest singlet excited state is of ^1^ *A*_1_ symmetry. We performed UNO-SLG based calculations in cc-pVXZ basis sets (X = T,Q,5) to obtain adiabatic singlet–triplet splittings, using geometries optimized by CASPT2 at the aug-cc-pVTZ level, as given in ref 79. Apart from the core geminal, all (three) other geminals are correlated in the reference. For the ground state, UNO-based SLG has a pure triplet solution.

A common feature of the SLG-F12 variants is that total energy (collected in Table 6) convergences faster than for SLG-PT2, both for the variationally optimized case and the SP-fixed amplitudes. For these cases, stepping from TZ to QZ basis yields a difference of about 2 mE_h_, while the change from QZ to 5Z basis is 1 order of magnitude smaller. This is mainly attributed to the change in the SLG reference energy. As observed before, the limits are different, with variant E + SI + I recovering the most amount, 85–87% of the SLG-PT2 correlation energy. Unsurprisingly, the geminal-based approaches that use the SP-Ansatz yield higher energies than their variational versions, providing 78% of the SLG-PT2 energy.

**Table 6:** Total Energy (in E_h_) of the ^3^ *B*_1_ Ground State of Methylene in cc-pVXZ Basis Sets, Obtained by SLG-Based Methods^a^

	${Ĥ}_{ext2}$			${Ĥ}_{ext1}$		
	cc-pVTZ	cc-pVQZ	cc-pV5Z	cc-pVTZ	cc-pVQZ	cc-pV5Z
SLG	–38.92901	–38.93125	–38.93180	–38.92901	–38.93125	–38.93180
PT2	–39.03717	–39.04601	–39.04878	–39.05311	–39.06412	–39.06788
E	–39.00449	–39.00614	–39.00676	–39.02217	–39.02367	–39.02432
E + SI + I′	–39.02376	–39.02594	–39.02658	–39.04317	–39.04519	–39.04586
E + SI + I	–39.02878	–39.03106	–39.03171	–39.04831	–39.05044	–39.05111
E (SP)	–39.00125	–39.00301	–39.00364	–39.01529	–39.01673	–39.01734
E + SI + I′ (SP)	–39.01881	–39.02086	–39.02147	–39.03284	–39.03458	–39.03517
E + SI + I (SP)	–39.02157	–39.02360	–39.02420	–39.03560	–39.03732	–39.03789

a See text and legend of Table 2 for abbreviations.

The singlet–triplet energy differences listed in Table 7 give better insight into the performance of our model. Without dynamic correlation, UNO-SLG overestimates the gap. Conventional PT2 improves the gaps, especially if the 1-body zero-order Hamiltonian is used for the virtual orbitals $\left({Ĥ}_{ext1}\right)$ . With the exception of the E + SI variant (Table S6 in Supporting Information), SLG-F12 methods also shift the gaps of the SLG reference to the correct direction. If semi-internal configurations are present in the F12 component, then variant E + SI + I′ with optimized amplitudes yields the best results: compared to experimental data, the error is 0.06–0.04 eV and 0.02–0.01 eV for the 2-particle and 1-particle external zero-order Hamiltonians, respectively. The latter yields a gap of similar quality than the more sophisticated MRCI methods which use a CAS with 6 active orbitals. For methylene, SLG-F12 with E + SI + I′ provides more accurate gaps than CASPT2 with the same active space as MRCI. We mention that CASPT2 and CASPT2-F12 yield much better excitation energies when a larger active space is used (15 active orbitals, cf. ref 79). However, we chose the smaller CAS for the comparison, since our UNO-SLG reference has also 6 active orbitals (but a much more compact CI space than CAS­(6,6)). SLG-F12 variant E + SI + I′ with the 2-electron external Hamiltonian is comparable to the SF-OD spin-flip approach,^101^ while the same variant using ${Ĥ}_{ext1}$ is closer to the CASSCF­(2,2)-based block-correlated CC (CAS-BCCC4) values.^104^

**Table 7:** Adiabatic Gap (*E*(^1^ *A*_1_) – *E*(^3^ *B*_1_) in eV) of Methylene in cc-pVXZ Basis Sets, Obtained by SLG-Based Methods

	${Ĥ}_{ext2}$			${Ĥ}_{ext1}$		
	cc-pVTZ	cc-pVQZ	cc-pV5Z	cc-pVTZ	cc-pVQZ	cc-pV5Z
SLG	0.601	0.586	0.582	0.601	0.586	0.582
PT2	0.540	0.508	0.496	0.480	0.442	0.427
E	0.418	0.412	0.408	0.354	0.350	0.347
E + SI + I′	0.466	0.453	0.449	0.421	0.410	0.407
E + SI + I	0.517	0.505	0.501	0.472	0.462	0.459
E (SP)	0.416	0.408	0.405	0.359	0.354	0.351
E + SI + I′ (SP)	0.515	0.504	0.500	0.458	0.450	0.446
E + SI + I (SP)	0.546	0.535	0.531	0.488	0.480	0.476
SF-OD^a^	0.483	0.451				
CAS-BCCC4^b^	0.441	0.398				

	cc-pVDZ-F12	cc-pVTZ-F12	cc-pVQZ-F12
CASPT2^c^	0.615	0.583	0.571
CASPT2-F12^c^	0.549	0.552	0.553
MRCI^d^	0.438	0.390	0.379
MRCI-F12^d^	0.405	0.374	0.370
MRCI-[2]_*R*12_ ^e^	0.387	0.363	0.361
expt.^f^	0.406		

a Reference 101.

b Reference 104.

c Reference 79.

d Reference 53.

e Reference 85.

f Reference 105 based on ref 106.

An interesting result is obtained when only fully external configurations are included in the explicitly correlated correction (variant E): singlet–triplet splittings are surprisingly accurate with the 2-body zero-order external Hamiltonian. Since Table 6 clearly shows that this Ansatz seems to lack a substantial amount of correlation, the quality of this gap might by a consequence of error cancellation. Generally, variants employing the SP-Ansatz perform less accurately compared to their optimized counterparts. For the E + SI + I(′) variants, this means a 0.02–0.05 eV difference between variational and SP-fixed singlet–triplet gaps.

### 4.4 O_3_

Our final example is ozone, which is known to be a particularly interesting system for MR methods due to the intricate interplay between static and dynamic correlation. Vertical excitation energy of the ^3^ *B*_2_ state was calculated based on spin-pure UNO-SLG solutions, at the experimental geometry^107^ of the ^1^ *A*_1_ ground state, which has *C*_2*v*_ symmetry. For both singlet and triplet states, there are 6 two-dimensional geminals in the reference. Vertical gaps are compared to results from SLG-PT2, various single- and multireference methods as well as experiment.

Overall trends regarding the total energies (Table 8) are analogous to the previous examples. Compared to PT2, variant E + SI + I with optimized amplitudes yields 90 and 92% of the correlation energy for the two partitionings (cc-pV5Z basis). Frozen core SLG-F12 energies converge faster than conventional PT2, with energy differences about 11–13 mE_h_ (T → Q) and 4–5 mE_h_ (Q → 5) for the explicitly correlated methods. SP-fixed results are higher than their variational counterparts by about 60–70 mE_h_ (E + SI + I′ and E + SI + I). Total energy decreases as more configurations are included in the F12 component (E, E + SI′ etc.), even though the variational degrees of freedom remain the same.

**Table 8:** Total Energy (in E_h_) of the ^1^ *A*_1_ Ground State of Ozone in cc-pVXZ Basis Sets, Obtained with SLG-Based Methods^a^

	${Ĥ}_{ext2}$			${Ĥ}_{ext1}$		
	cc-pVTZ	cc-pVQZ	cc-pV5Z	cc-pVTZ	cc-pVQZ	cc-pV5Z
SLG	–224.43554	–224.45323	–224.45794	–224.43554	–224.45323	–224.45794
PT2	–225.02195	–225.08736	–225.11121	–225.05702	–225.13174	–225.16079
E	–224.75717	–224.76503	–224.76946	–224.80866	–224.81457	–224.81884
E + SI + I′	–225.00982	–225.02245	–225.02724	–225.06969	–225.08047	–225.08515
E + SI + I	–225.03019	–225.04321	–225.04806	–225.09031	–225.10147	–225.10621
E (SP)	–224.72887	–224.74252	–224.74758	–224.79002	–224.80083	–224.80559
E + SI + I (SP)	–224.94793	–224.96335	–224.96782	–225.00909	–225.02166	–225.02582
E + SI + I′ (SP)	–224.96131	–224.97668	–224.98109	–225.02246	–225.03499	–225.03909

a For notation, refer to Table 2.

Concerning the adiabatic gaps presented in Table 9, we observe similar tendencies as before. Without dynamic correlation, SLG gives energy differences that are too small. Inclusion of dynamic effects via PT2 or F12 improves the quality of the gaps. Singlet–triplet splittings by variants including semi-internal as well as fully internal configurations (i.e., E + SI + I(′)) with optimized amplitudes fall closest to standard SLG-PT2, and they are closest to SR linear response CCSD (CCSD-LR) and Fock space based MR CCSD (IH-FS-CCSD) results. Switching to SP-fixed amplitudes decreases the gaps by 0.2–0.25 eV for these SLG-F12 variants. Inclusion of semi-internal excitations without the presence of fully internals (E + SI) yields once again inadequate singlet–triplet splittings (Table S8 in Supporting Information). In contrast with the previous examples, the simplest scheme employing only the doubly external configurations (E) performs even worse than E + SI or SLG by itself.

**Table 9:** Vertical Excitation Energy (*E*(^3^ *B*_2_) – *E*(^1^ *A*_1_) in eV) of Ozone in cc-pVXZ Basis Sets, Obtained by SLG-Based Methods with Frozen Core Calculations

	${Ĥ}_{ext2}$			${Ĥ}_{ext1}$		
	cc-pVTZ	cc-pVQZ	cc-pV5Z	cc-pVTZ	cc-pVQZ	cc-pV5Z
SLG	0.900	0.902	0.901	0.900	0.902	0.901
PT2	1.427	1.440	1.446	1.421	1.434	1.440
E	0.872	0.873	0.871	0.871	0.872	0.870
E + SI + I′	1.317	1.325	1.328	1.320	1.329	1.332
E + SI + I	1.438	1.447	1.449	1.443	1.451	1.454
E (SP)	0.874	0.875	0.873	0.870	0.871	0.870
E + SI + I′ (SP)	1.103	1.106	1.105	1.100	1.103	1.102
E + SI + I (SP)	1.172	1.175	1.174	1.169	1.172	1.171
MRCI^a^	1.701					

	cc-pCVDZ	cc-pCVTZ	cc-pCVQZ
CCSD-LR^b^	1.421	1.398	1.402
CCSDT-LR^b^	1.716	1.737	
Mk-MRCCSD-LR^b^	1.920	1.908	1.919

	aug-cc-pVTZ	aug-cc-pVQZ	aug-cc-pV5Z
EOM-CCSD^c^	1.60	1.62	1.59
STEOM-CCSD^c^	1.58	1.66	
IH-FS-CCSD^c^	1.46	1.54	
expt.^d^	1.29; 1.43		

a Reference 108.

b Reference 109.

c Reference 110.

d Reference 111.

## 5 Conclusion

The explicitly correlated correction of a strongly orthogonal geminals’ reference was introduced in this paper. The F12 component is obtained perturbatively by means of a Dyall-like partitioning of the Hamiltonian. Aiming at capturing dynamic correlation without the presence of other corrections, different SLG-F12 schemes were tested, differing in either the form of ${Ĥ}^{\left(0\right)}$ or the type of excited configurations entering the explicitly correlated component. Both variationally optimized and SP-fixed F12 amplitudes were implemented for the theory.

Incorporating the explicitly correlated correction enhances convergence of total energies with respect to the basis size compared to conventional SLG-based PT2. As anticipated, the limits are not the same, however. To obtain total energies closest in quality to SLG-PT2, inclusion of semi-internal and fully internal excitations is required (variant E + SI + I), recovering 85–90% of the SLG-PT2 correlation energy. Our results show that apart from their contribution to the total energy, the effect of the fully internal excitations on the balanced characterization of the different states is significant, as is apparent from the singlet–triplet gaps presented in this work. Inclusion of these internal configurations is important for a good description of both states. Therefore, variant E + SI, which has the worst performance, is not recommended. Based on the quality of the S-T energy differences, inclusion of semi-internals in the Ansatz is justified but only if fully internals are also present in the theory.

In several cases, the simplest variant, which includes only the doubly external configurations in the F12 part combined with the 2-body external component of ${Ĥ}^{\left(0\right)}$ yields rather accurate gaps, which is surprising if one looks at the absolute energies by this method. The quality of the gaps is therefore a consequence of errors canceling each other out. Still, for a quick assessment of energy splittings, even this simple framework might be enough. However, to obtain more reliable results, SI + I excitations should be also included, which brings a neglectable increase in computational effort.

Whether we use optimized or SP-fixed amplitudes is not inconsequential. While variants E and E + SI are less sensitive to this issue, the schemes including internal excitations can perform differently for the two sets of amplitudes, differences between gaps with the two sets of amplitudes being 0.05–0.2 eV. Apart from the NH molecule, using the SP-amplitudes is not preferred. Though this fact is probably due to a mix of different effects which can be hard to identify, the lack of conventional orbital expansions certainly plays a role, since in conventional explicitly correlated approaches, the SP-Ansatz is remarkably efficient. Whether the inclusion of singles in the Ansatz or orbital optimization will change this feature prompts further investigations along this line.

The best performing variants are those which include as much as possible in the explicitly correlated correction, i.e., E + SI + I′ and E + SI + I, using the variationally optimized amplitudes. Overall, employing only the 1-particle operator of the external part of ${Ĥ}^{\left(0\right)}$ seems to give the better performance. Singlet–triplet gaps by this variant, particularly E + SI + I′, are remarkably accurate. This is especially compelling if we take into account the simplicity and compactness of our model. Despite its modest parametrization, it can provide a reliable description of static and to a great extent also the dynamic correlation effects, hence recovering a substantial amount of the SLG-PT2 correlation energy. Although the compact SLG-F12 Ansatz can not account for all the correlation effects, it can treat lowest lying singlet and triplet states of small molecules in a balanced manner. Accordingly, the SLG-F12 model can be used as a screening method thanks to its low computational cost.

## Supplementary Material



## Figures and Tables

**A1 tbl10:**
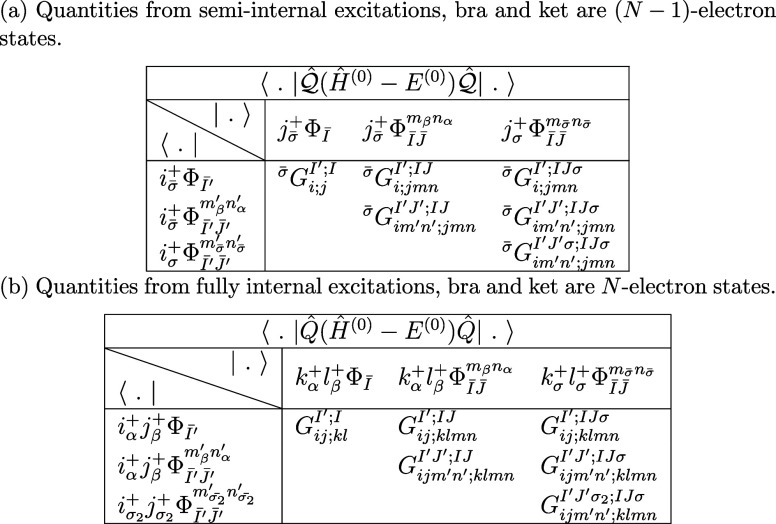
Notation for Matrix Elements of the Operator ${Ĥ}^{\left(0\right)}-E^{\left(0\right)}$ between SLG-Based States

**A2 tbl11:**
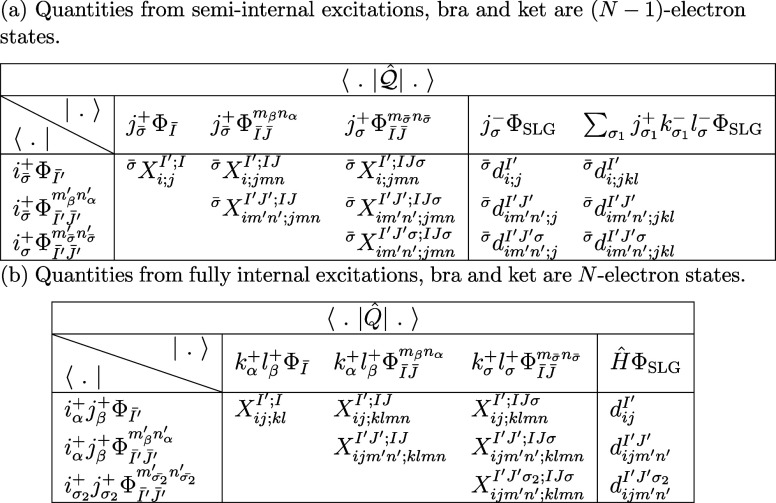
Notation for Overlap Matrix Elements between SLG-Based States

## Footnotes

a The term geminal is extensively used in the explicitly correlated literature: for the *r*_12_-containing two-electron functions as well as for the quasi-double excitations generated from the reference through an F12 operator. Both of these obviously differ from the geminals in the SLG Ansatz.

## Appendix

In this section, we adopt the Einstein convention for summation over repeated indices. If a summation is restricted to e.g., a geminal subspace, then it is written explicitly. Matrix elements involving F12 integrals were evaluated by applying the CABS approach^93^ followed by SA. First, we define intermediates stemming from fully external excitations as

$$\begin{array}{l} \begin{array}{l} \begin{array}{l} \begin{array}{l} X_{k^{\prime }l^{\prime }}^{kl}=F_{k^{\prime }l^{\prime }}^{xy}F_{xy}^{kl}\overset{SA}{=}{\left(f_{12}^{2}\right)}_{k^{\prime }l^{\prime }}^{kl}-F_{k^{\prime }l^{\prime }}^{pq}F_{pq}^{kl}+F_{k^{\prime }l^{\prime }}^{ab}F_{ab}^{kl}\\ V_{k^{\prime }l^{\prime }}^{ij}=F_{k^{\prime }l^{\prime }}^{xy}g_{xy}^{ij}\overset{SA}{=}{\left(f_{12}g_{12}\right)}_{k^{\prime }l^{\prime }}^{ij}-F_{k^{\prime }l^{\prime }}^{pq}g_{pq}^{ij}+F_{k^{\prime }l^{\prime }}^{ab}g_{ab}^{ij} \end{array}\\ P_{k^{\prime }l^{\prime }}^{kl}=F_{k^{\prime }l^{\prime }}^{x^{\prime }y^{\prime }}g_{x^{\prime }y^{\prime }}^{xy}F_{xy}^{kl}\overset{SA}{=}{\left(f_{12}g_{12}f_{12}\right)}_{k^{\prime }l^{\prime }}^{kl}-{\left(f_{12}g_{12}\right)}_{k^{\prime }l^{\prime }}^{pq}F_{pq}^{kl}+F_{k^{\prime }l^{\prime }}^{pq}{\left(g_{12}f_{12}\right)}_{pq}^{kl} \end{array}\\ +{\left(f_{12}g_{12}\right)}_{k^{\prime }l^{\prime }}^{ab}F_{ab}^{kl}+F_{k^{\prime }l^{\prime }}^{ab}{\left(g_{12}f_{12}\right)}_{ab}^{kl}\\ +F_{k^{\prime }l^{\prime }}^{pq}g_{pq}^{rs}F_{rs}^{kl}-F_{k^{\prime }l^{\prime }}^{pq}g_{pq}^{ab}F_{ab}^{kl}-F_{k^{\prime }l^{\prime }}^{ab}g_{ab}^{pq}F_{pq}^{kl}+F_{k^{\prime }l^{\prime }}^{ab}g_{ab}^{cd}F_{cd}^{kl} \end{array}\\ {\left(B^{\sigma }\right)}_{k^{\prime }l^{\prime }}^{kl}=F_{k^{\prime }l^{\prime }}^{xy^{\prime }}h_{y^{\prime }y}^{\sigma }F_{xy}^{kl}\overset{SA}{=}\Lambda_{k^{\prime }l^{\prime }}^{kl}+\frac{1}{2}\left\{{\left(f_{12}^{2}\right)}_{k^{\prime }l^{\prime }}^{kp}H_{pl}+H_{l^{\prime }p}{\left(f_{12}^{2}\right)}_{k^{\prime }p}^{kl}\right\}\\ -F_{k^{\prime }l^{\prime }}^{pq}H_{qr}F_{\mathrm{pr}}^{kl}+F_{k^{\prime }l^{\prime }}^{ab}h_{bc}^{\sigma }F_{ac}^{kl} \end{array}$$
A.1

To arrive at the expression for $B^{\sigma }$ given in eq A.1, approximation C^51^ was used, along with the definitions

A.2
$$\begin{array}{l} H_{\kappa \lambda }=h_{\kappa \lambda }+\sum_{I=1}^{N/2}\sum_{ij}^{\left(I\right)}g_{\kappa i}^{\lambda j}\gamma_{ij}\\ k_{\kappa \lambda }^{\sigma }=H_{\kappa \lambda }-h_{\kappa \lambda }^{\sigma }\\ \mathrm{\Lambda ̂}=\frac{1}{2}\left(f^{\prime }{\left(r_{12}\right)}^{2}\right) \end{array}$$

with *f*′(*t*) denoting the derivative of the function *f*(*t*). If *f*(*t*) = *t* (i.e., *f*_12_ = *r*_12_), then Λ̂ = 1/2, whereas if $f\left(t\right)=-\frac{1}{\gamma }\exp\left(-\gamma t\right)$ , then $\mathrm{\Lambda ̂}=\frac{1}{2}\exp\left(-2\gamma r_{12}\right)$ .

Semi-internal excitations lead to matrix elements which have partial wave expansions that break off after a finite l value. Thus within the CABS approach, they can be computed by changing a summation over {*x*} to summations over {*a*} and { p̃ } (with the latter indicating the complementary set). If SA is applied as well, then only summation over {*a*} remains

A.3
$$\begin{array}{l} \begin{array}{l} X_{k^{\prime }l^{\prime };i}^{kl;j}≔F_{k^{\prime }l^{\prime }}^{xj}F_{xi}^{kl}\overset{SA}{=}F_{k^{\prime }l^{\prime }}^{aj}F_{ai}^{kl}\\ {\left(B^{\sigma }\right)}_{k^{\prime }l^{\prime };i}^{kl;j}≔F_{k^{\prime }l^{\prime }}^{x^{\prime }j}h_{x^{\prime }x}^{\sigma }F_{xi}^{kl}\overset{SA}{=}F_{k^{\prime }l^{\prime }}^{aj}h_{ab}^{\sigma }F_{bi}^{kl} \end{array}\\ Y_{k^{\prime }l^{\prime }}^{ij}≔F_{k^{\prime }l^{\prime }}^{xj}h_{xi}\overset{SA}{=}F_{k^{\prime }l^{\prime }}^{aj}h_{ai}\\ Y_{k^{\prime }l^{\prime };i}^{kl;j}:=F_{k^{\prime }l^{\prime }}^{xj}g_{xi}^{kl}\overset{SA}{=}F_{k^{\prime }l^{\prime }}^{aj}g_{ai}^{kl} \end{array}$$

Finally, fully internal configurations generate expressions such as $F_{k^{\prime }l^{\prime }}^{ij}F_{im}^{kl}$ or $F_{k^{\prime }l^{\prime }}^{ij}g_{ij}^{mn}F_{mn}^{kl}$ . They can be identified as intermediate matrix elements, but since any summation is simply over (a subset of) the partially occupied orbitals, these do not necessitate any manipulation and can be evaluated in an ordinary fashion and we do not give them in detail.

Notation for matrix elements which only involve orbitals constituting the SLG reference are given in Tables 10 and 11. Their explicit form can be derived based on ref 40 hence we omit their detailed expressions. Projector Q̂ is based on Q̂ by essentially having the same effect on the fully internal (*N* – 1)-electron part of a semi-internal excited state as Q̂ . Namely, $\mathrm{Q̂}x_{\sigma }^{+}\phi_{N-1}=x_{\sigma }^{+}\mathrm{Q̂}\phi_{N-1}$ . Having defined intermediate quantities above, the blocks contributing to the Hylleraas-functional are as follows.

A.4a
$$\left\langle \Phi_{\overset{®}{I^{\prime }\;}}\left|F_{\alpha ,\beta }^{k^{\prime }l^{\prime }-}\mathrm{Q̂}\left({Ĥ}^{\left(0\right)}-E^{\left(0\right)}\right)\mathrm{Q̂}F_{\alpha ,\beta }^{kl+}\right|\Phi_{\mathrm{I̅}}\right\rangle =$$

A.4b
$$=\delta_{I^{\prime }I}\left\{P_{k^{\prime }l^{\prime }}^{kl}+{\left(B^{\beta }\right)}_{k^{\prime }l^{\prime }}^{kl}+{\left(B^{\alpha }\right)}_{l^{\prime }k^{\prime }}^{lk}-E_{I}X_{k^{\prime }l^{\prime }}^{kl}\right\}$$

+δI′IXk′l′;ikl;jGj;iI;Iβ+Xj;iI;Iβ(Bα)k′l′;ikl;j+Xl′k′;ilk;jGj;iI;Iα+Xj;iI;Iα(Bβ)l′k′;ilk;j}
A.4c

A.4d
$$+\delta_{I^{\prime }I}F_{k^{\prime }l^{\prime }}^{i^{\prime }j^{\prime }}G_{i^{\prime }j^{\prime };ij}^{I^{\prime };I}F_{ij}^{kl}$$

Terms in eq A.4b stem from the doubly external excitations. Semi-internal contributions are given by eq A.4c, while the fully internal excitations which are not projected out provide the terms in eq A.4d. Depending on the exact form of Q̂ , these latter two contributions may become zero. Further matrix elements include

$$\left\langle \Phi_{\overset{®}{I^{\prime }\;}\overset{®}{J^{\prime }\;}}^{m_{\beta }^{\prime }n_{\alpha }^{\prime }}\left|F_{\alpha ,\beta }^{k^{\prime }l^{\prime }-}\mathrm{Q̂}\left({Ĥ}^{\left(0\right)}-E^{\left(0\right)}\right)\mathrm{Q̂}F_{\alpha ,\beta }^{kl+}\right|\Phi_{\mathrm{I̅}\mathrm{J̅}}^{m_{\beta }n_{\alpha }}\right\rangle =$$

$$=\delta_{I^{\prime }I}\delta_{J^{\prime }J}\left\{\delta_{n^{\prime }n}\delta_{m^{\prime }m}\left(P_{k^{\prime }l^{\prime }}^{kl}+{\left(B^{\beta }\right)}_{k^{\prime }l^{\prime }}^{kl}+{\left(B^{\alpha }\right)}_{l^{\prime }k^{\prime }}^{lk}-\left(E_{I}+E_{J}\right)X_{k^{\prime }l^{\prime }}^{kl}\right)+X_{k^{\prime }l^{\prime }}^{kl}\left(\delta_{m^{\prime }m}h_{n^{\prime }n}^{eff,\alpha }+\delta_{n^{\prime }n}h_{m^{\prime }m}^{eff,\beta }\right)\right\}$$

+δI′IδJ′J{Xk′l′;ikl;jGjm′n′;imnI′J′;IJβ+Xjm′n′;imnI′J′;IJβ(Bα)k′l′;ikl;j

+Xl′k′;ilk;jGjm′n′;imnI′J′;IJα+Xjm′n′;imnI′J′;IJα(Bβ)l′k′;ilk;j}

A.5
$$+\left(\delta_{I^{\prime }I}\delta_{J^{\prime }J}+\delta_{I^{\prime }J}\delta_{J^{\prime }I}\right)F_{k^{\prime }l^{\prime }}^{i^{\prime }j^{\prime }}G_{i^{\prime }j^{\prime }m^{\prime }n^{\prime };ijmn}^{I^{\prime }J^{\prime };IJ}F_{ij}^{kl}$$

Similarly, we get

$$\left\langle \Phi_{\overset{®}{I^{\prime }\;}\overset{®}{J^{\prime }\;}}^{m_{\beta }^{\prime }n_{\alpha }^{\prime }}\left|F_{\alpha ,\beta }^{k^{\prime }l^{\prime }-}\mathrm{Q̂}\left({Ĥ}^{\left(0\right)}-E^{\left(0\right)}\right)\mathrm{Q̂}F_{\alpha ,\alpha }^{kl+}\right|\Phi_{\mathrm{I̅}\mathrm{J̅}}^{m_{\beta }n_{\beta }}\right\rangle =$$

=(δI′IδJ′J+δI′JδJ′I){X̅k′l′;ikl;jGjm′n′;imnI′J′;IJαβ+Xjm′n′;imnI′J′;IJαβ(Bα®)k′l′;ikl;j

A.6
$$+F_{k^{\prime }l^{\prime }}^{i^{\prime }j^{\prime }}G_{i^{\prime }j^{\prime }m^{\prime }n^{\prime };ijmn}^{I^{\prime }J^{\prime };IJ\alpha }F_{ij}^{kl}\}$$

where intermediate quantities with bars are antisymmetrized versions of those in eq A.3, e.g., ${\mathrm{X̅}}_{k^{\prime }l^{\prime };i}^{kl;j}=X_{k^{\prime }l^{\prime };i}^{kl;j}-X_{k^{\prime }l^{\prime };i}^{lk;j}$ . Analogously

$$\left\langle \Phi_{\overset{®}{I^{\prime }\;}\overset{®}{J^{\prime }\;}}^{m_{\beta }^{\prime }n_{\alpha }^{\prime }}\left|F_{\alpha ,\beta }^{k^{\prime }l^{\prime }-}\mathrm{Q̂}\left({Ĥ}^{\left(0\right)}-E^{\left(0\right)}\right)\mathrm{Q̂}F_{\beta ,\beta }^{kl+}\right|\Phi_{\mathrm{I̅}\mathrm{J̅}}^{m_{\alpha }n_{\alpha }}\right\rangle =$$

=(δI′IδJ′J+δI′JδJ′I){X̅l′k′;ikl;jGjm′n′;imnI′J′;IJβα+Xjm′n′;imnI′J′;IJβα(Bβ®)l′k′;ikl;j

A.7
$$+F_{k^{\prime }l^{\prime }}^{i^{\prime }j^{\prime }}G_{i^{\prime }j^{\prime }m^{\prime }n^{\prime };ijmn}^{I^{\prime }J^{\prime };IJ\beta }F_{ij}^{kl}\}$$

while

$$\left\langle \Phi_{\overset{®}{I^{\prime }\;}\overset{®}{J^{\prime }\;}}^{m_{\mathrm{\sigma ̅}}^{\prime }n_{\mathrm{\sigma ̅}}^{\prime }}\left|F_{\sigma ,\sigma }^{k^{\prime }l^{\prime }-}\mathrm{Q̂}\left({Ĥ}^{\left(0\right)}-E^{\left(0\right)}\right)\mathrm{Q̂}F_{\sigma ,\sigma }^{kl+}\right|\Phi_{\mathrm{I̅}\mathrm{J̅}}^{m_{\mathrm{\sigma ̅}}n_{\mathrm{\sigma ̅}}}\right\rangle$$

$$=\delta_{I^{\prime }I}\delta_{J^{\prime }J}\{\delta_{n^{\prime }n}\delta_{m^{\prime }m}\left({\mathrm{P̅}}_{k^{\prime }l^{\prime }}^{kl}+{\left(\overset{®}{B^{\sigma }\;}\right)}_{k^{\prime }l^{\prime }}^{kl}+{\left(\overset{®}{B^{\sigma }\;}\right)}_{l^{\prime }k^{\prime }}^{lk}-\left(E_{I}+E_{J}\right){\mathrm{X̅}}_{k^{\prime }l^{\prime }}^{kl}\right)+{\mathrm{X̅}}_{k^{\prime }l^{\prime }}^{kl}\left(\delta_{m^{\prime }m}h_{n^{\prime }n}^{eff,\mathrm{\sigma ̅}}+\delta_{n^{\prime }n}h_{m^{\prime }m}^{eff,\mathrm{\sigma ̅}}\right)$$

+(X̅k′l′;ikl;j+X̅l′k′;ilk;j)Gjm′n′;imnI′J′σ;IJσσ̅+Xjm′n′;imnI′J′σ;IJσσ̅((Bσ)k′l′;ikl;j+(Bσ)l′k′;ilk;j)

A.8
$$+F_{k^{\prime }l^{\prime }}^{i^{\prime }j^{\prime }}G_{i^{\prime }j^{\prime }m^{\prime }n^{\prime };ijmn}^{I^{\prime }J^{\prime }\sigma ;IJ\sigma }F_{ij}^{kl}\}$$

with bar meaning antisymmetrization, e.g., ${\mathrm{P̅}}_{k^{\prime }l^{\prime }}^{kl}=P_{k^{\prime }l^{\prime }}^{kl}-P_{k^{\prime }l^{\prime }}^{lk}$ . Finally

A.9
$$\left\langle \Phi_{\overset{®}{I^{\prime }\;}\overset{®}{J^{\prime }\;}}^{m_{\beta }^{\prime }n_{\beta }^{\prime }}\left|F_{\alpha ,\alpha }^{k^{\prime }l^{\prime }-}\mathrm{Q̂}\left({Ĥ}^{\left(0\right)}-E^{\left(0\right)}\right)\mathrm{Q̂}F_{\beta ,\beta }^{kl+}\right|\Phi_{\mathrm{I̅}\mathrm{J̅}}^{m_{\alpha }n_{\alpha }}\right\rangle =\delta_{I^{\prime }I}\delta_{J^{\prime }J}F_{k^{\prime }l^{\prime }}^{i^{\prime }j^{\prime }}G_{i^{\prime }j^{\prime }m^{\prime }n^{\prime };ijmn}^{I^{\prime }J^{\prime }\alpha ;IJ\beta }F_{ij}^{kl}$$

Terms generating the linear part of the Hylleraas-functional are

$$\left\langle \Phi_{\mathrm{I̅}}\left|F_{\alpha ,\beta }^{kl-}\mathrm{Q̂}Ĥ\right|SLG\right\rangle =-\sum_{ij}^{\left(I\right)}C_{ji}V_{kl}^{ij}$$

+di′;iIβYklii′+di′;ijj′IβYkl;ij′j;i′−di′;iIαYlkii′−di′;ijj′IαYlk;ij′j;i′+dijIFklij
A.10

and

$$\left\langle \Phi_{\mathrm{I̅}\mathrm{J̅}}^{m_{\beta }n_{\alpha }}\left|F_{\alpha ,\beta }^{kl-}\mathrm{Q̂}Ĥ\right|SLG\right\rangle =\sum_{i}^{\left(I\right)}\sum_{j}^{\left(J\right)}C_{mi}C_{jn}V_{kl}^{ij}$$

+di′mn;iIJβYklii′+di′mn;ijj′IJβYkl;ij′j;i′−di′mn;iIJα−Ylkii′di′mn;ijj′IJαYlk;ij′j;i′+dijmnIJFklij
A.11

Analogously

$$\left\langle \Phi_{\mathrm{I̅}\mathrm{J̅}}^{m_{\beta }n_{\beta }}\left|F_{\alpha ,\alpha }^{kl-}\mathrm{Q̂}Ĥ\right|SLG\right\rangle =\sum_{i}^{\left(I\right)}\sum_{j}^{\left(J\right)}C_{mi}C_{nj}{\mathrm{V̅}}_{kl}^{ji}$$

+di′mn;iIJαβY̅klii′+di′mn;ijj′IJαβY̅kl;ij′j;i′+dijmnIJαFklij
A.12

and finally

$$\left\langle \Phi_{\mathrm{I̅}\mathrm{J̅}}^{m_{\alpha }n_{\alpha }}\left|F_{\beta ,\beta }^{kl-}\mathrm{Q̂}Ĥ\right|SLG\right\rangle =\sum_{i}^{\left(I\right)}\sum_{j}^{\left(J\right)}C_{im}C_{jn}{\mathrm{V̅}}_{kl}^{ji}$$

+di′mn;iIJβαY̅klii′+di′mn;ijj′IJβαY̅kl;ij′j;i′+dijmnIJβFklij
A.13

In eqs A.12, A.13 antisymmetrized quantities appear as ${\mathrm{V̅}}_{kl}^{ji}=V_{kl}^{ji}-V_{lk}^{ji}$ , whereas ${\mathrm{Y̅}}_{kl}^{ii^{\prime }}=Y_{kl}^{ii^{\prime }}-Y_{lk}^{ii^{\prime }}$ and ${\mathrm{Y̅}}_{kl;i}^{j^{\prime }j;i^{\prime }}=Y_{kl;i}^{j^{\prime }j;i^{\prime }}-Y_{lk;i}^{j^{\prime }j;i^{\prime }}$ .

## Notes

SLG-F12 total energies and singlet–triplet gaps by the variant denoted “E + SI” (frozen core results); SLG-F12 total energies and gaps obtained by fully correlated calculations (all variants) (PDF)
