# Supplementary material for: Combining Strongly Orthogonal Geminals with Explicitly Correlated Corrections
Source: J Chem Theory Comput. 2025 Aug 5;21(16):7765–81. doi: 10.1021/acs.jctc.5c00264 (PMC12393080; doi:10.1021/acs.jctc.5c00264)
Supplement: Supplementary file 1 [file ct5c00264_si_001.pdf]

Supporting Information: Combining strongly orthogonal geminals with explicitly correlated corrections

Zs. É. Mihálka* and J. Noga*

Department of Inorganic Chemistry, Faculty of Natural Sciences, Comenius University in Bratislava, Mlynská dolina, Ilkovičova 6, 842 15 Bratislava 4, Slovakia

E-mail: zsuzsanna.mihalka@uniba.sk; jozef.noga@uniba.sk

Here we provide results of fully correlated calculations for O_2, NH, CH_2 and O_3, as well as total energies and gaps obtained by the SLG-F12 variant denoted E+SI.

1 O_2

Table S1: Total energy (in E_h) of the $^3\Sigma$ ground state of O_2 in cc-pVXZ and cc-pCVXZ basis sets with the E+SI variant of SLG-F12. For notation, see Table 2 of the main text.

	core and valence electrons correlated					
	$\hat{H}_{\text{ext}_2}$			$\hat{H}_{\text{ext}_1}$		
	cc-pVTZ	cc-pVQZ	cc-pV5Z	cc-pVTZ	cc-pVQZ	cc-pV5Z
E+SI	−150.16674	−150.13301	−150.13202	−150.21686	−150.18032	−150.17816
E+SI(SP)	−149.98921	−149.98843	−149.98570	−150.05281	−150.04360	−150.03960
	valence electrons correlated					
E+SI	−149.99892	−150.01353	−150.01887	−150.03799	−150.05254	−150.05824
E+SI(SP)	−149.97142	−149.98682	−149.99166	−150.01079	−150.02461	−150.02940
	all electrons correlated					
	cc-pCVTZ	cc-pCVQZ	cc-pCV5Z	cc-pCVTZ	cc-pCVQZ	cc-pCV5Z
E+SI	−150.12043	−150.12670	−150.13102	−150.16708	−150.17219	−150.17684
E+SI (SP)	−149.98435	−149.99115	−149.99513	−150.03828	−150.04412	−150.04818

Table S2: Adiabatic singlet-triplet gap ($E(^1\Delta) - E(^3\Sigma)$, in eV) of O_2 in cc-pVXZ basis sets.

	core and valence electrons correlated					
	$\hat{H}_{\text{ext2}}$			$\hat{H}_{\text{ext1}}$		
	cc-pVTZ	cc-pVQZ	cc-pV5Z	cc-pVTZ	cc-pVQZ	cc-pV5Z
PT2	1.102	1.070	1.058	1.083	1.047	1.031
E	1.183	1.194	1.194	1.150	1.167	1.168
E+SI	1.386	1.397	1.400	1.362	1.376	1.379
E+SI+I’	1.095	1.111	1.111	1.076	1.081	1.082
E+SI+I	1.056	1.087	1.087	1.052	1.054	1.055
E(SP)	1.182	1.197	1.200	1.144	1.162	1.165
E+SI(SP)	1.364	1.384	1.389	1.326	1.349	1.354
E+SI+I’(SP)	1.207	1.221	1.225	1.169	1.186	1.190
E+SI+I(SP)	1.205	1.219	1.222	1.167	1.184	1.188
	valence electrons correlated					
E+SI	1.373	1.385	1.386	1.350	1.365	1.366
E+SI(SP)	1.383	1.396	1.399	1.345	1.360	1.364

2 NH

Table S3: Total energy (in E_h) of the $^3\Sigma$ ground state of NH in cc-pVXZ basis sets, obtained by SLG-based methods. For notation, see Table 2 in main text.

	core and valence electrons correlated					
	$\hat{H}_{\text{ext2}}$			$\hat{H}_{\text{ext1}}$		
	cc-pVTZ	cc-pVQZ	cc-pV5Z	cc-pVTZ	cc-pVQZ	cc-pV5Z
PT2	−55.11263	−55.14073	−55.15061	−55.12849	−55.15974	−55.17118
E	−55.12893	−55.11247	−55.11123	−55.15102	−55.13368	−55.13186
E+SI	−55.15723	−55.14230	−55.14129	−55.18108	−55.16521	−55.16367
E+SI+I’	−55.15756	−55.14265	−55.14163	−55.18141	−55.16556	−55.16402
E+SI+I	−55.16680	−55.15208	−55.15107	−55.19077	−55.17512	−55.17359
E (SP)	−55.07879	−55.07309	−55.07039	−55.10634	−55.09714	−55.09398
E+SI (SP)	−55.10384	−55.09806	−55.09494	−55.13138	−55.12211	−55.11854
E+SI+I’ (SP)	−55.10409	−55.09831	−55.09520	−55.13164	−55.12237	−55.11880
E+SI+I (SP)	−55.10974	−55.10386	−55.10068	−55.13728	−55.12791	−55.12428
	valence electrons correlated					
E+SI	−55.08684	−55.09058	−55.09171	−55.10594	−55.10948	−55.11066
E+SI (SP)	−55.08152	−55.08541	−55.08652	−55.09753	−55.10082	−55.10191

Table S4: Adiabatic gap ($E(^1\Delta) - E(^3\Sigma)$ in eV) of NH in cc-pVXZ basis sets, obtained by SLG-based methods.

	core and valence electrons correlated					
	$\hat{H}_{\text{ext2}}$			$\hat{H}_{\text{ext1}}$		
	cc-pVTZ	cc-pVQZ	cc-pV5Z	cc-pVTZ	cc-pVQZ	cc-pV5Z
PT2	1.484	1.405	1.376	1.450	1.363	1.330
E	1.563	1.555	1.550	1.511	1.507	1.503
E+SI	1.903	1.881	1.872	1.879	1.859	1.851
E+SI+I'	1.394	1.357	1.346	1.359	1.325	1.315
E+SI+I	1.382	1.344	1.332	1.345	1.310	1.300
E (SP)	1.569	1.560	1.555	1.504	1.500	1.496
E+SI (SP)	1.893	1.869	1.860	1.828	1.809	1.801
E+SI+I' (SP)	1.596	1.577	1.570	1.532	1.516	1.510
E+SI+I (SP)	1.595	1.575	1.568	1.530	1.515	1.509
	valence electrons correlated					
E+SI	1.887	1.864	1.855	1.863	1.843	1.835
E+SI (SP)	1.894	1.871	1.862	1.829	1.810	1.802

3 CH_2

Table S5: Total energy (in E_h) of the $^3B_1$ ground state of CH_2 in cc-pVXZ basis sets, obtained by SLG-based methods. See main text and legend of Table 2 for abbreviations.

	core and valence electrons correlated					
	$\hat{H}_{\text{ext2}}$			$\hat{H}_{\text{ext1}}$		
	cc-pVTZ	cc-pVQZ	cc-pV5Z	cc-pVTZ	cc-pVQZ	cc-pV5Z
PT2	−39.04947	−39.07021	−39.07802	−39.06589	−39.08959	−39.09877
E	−39.06124	−39.05138	−39.05077	−39.08319	−39.07272	−39.07177
E+SI	−39.08151	−39.07251	−39.07202	−39.10519	−39.09558	−39.09479
E+SI+I'	−39.08179	−39.07279	−39.07228	−39.10548	−39.09586	−39.09506
E+SI+I	−39.08680	−39.07789	−39.07739	−39.11061	−39.10108	−39.10029
E (SP)	−39.02496	−39.02155	−39.02026	−39.04954	−39.04392	−39.04234
E+SI (SP)	−39.04246	−39.03917	−39.03771	−39.06703	−39.06155	−39.05978
E+SI+I' (SP)	−39.04270	−39.03940	−39.03793	−39.06728	−39.06178	−39.06000
E+SI+I (SP)	−39.04546	−39.04214	−39.04065	−39.07004	−39.06451	−39.06273
	valence electrons correlated					
E+SI	−39.02350	−39.02569	−39.02634	−39.04290	−39.04493	−39.04561
E+SI (SP)	−39.01857	−39.02064	−39.02126	−39.03260	−39.03436	−39.03495

Table S6: Adiabatic gap ($E(^1A_1) - E(^3B_1)$ in eV) of CH_2 in cc-pVXZ basis sets, obtained by SLG-based methods.

	core and valence electrons correlated					
	$\hat{H}_{\text{ext2}}$			$\hat{H}_{\text{ext1}}$		
	cc-pVTZ	cc-pVQZ	cc-pV5Z	cc-pVTZ	cc-pVQZ	cc-pV5Z
PT2	0.560	0.520	0.509	0.501	0.455	0.441
E	0.394	0.417	0.417	0.324	0.351	0.353
E+SI	0.656	0.674	0.672	0.614	0.634	0.635
E+SI+I'	0.449	0.465	0.464	0.399	0.419	0.420
E+SI+I	0.500	0.517	0.516	0.450	0.471	0.472
E (SP)	0.388	0.400	0.404	0.330	0.347	0.353
E+SI (SP)	0.618	0.625	0.628	0.560	0.572	0.576
E+SI+I' (SP)	0.489	0.498	0.503	0.431	0.446	0.451
E+SI+I (SP)	0.520	0.529	0.533	0.462	0.476	0.481
	valence electrons correlated					
E+SI	0.672	0.660	0.656	0.634	0.625	0.621
E+SI (SP)	0.644	0.631	0.626	0.587	0.576	0.572

4 O_3

Table S7: Total energy (in E_h) of the $^1A_1$ ground state of O_3 in cc-pVXZ basis sets, obtained by SLG-based methods. For notation, refer to Table 2.

	core and valence electrons correlated					
	$\hat{H}_{\text{ext2}}$			$\hat{H}_{\text{ext1}}$		
	cc-pVTZ	cc-pVQZ	cc-pV5Z	cc-pVTZ	cc-pVQZ	cc-pV5Z
PT2	−225.05799	−225.17162	−225.21411	−225.09391	−225.21898	−225.26769
E	−225.00211	−224.92665	−224.92004	−225.06800	−224.99079	−224.97906
E+SI	−225.17930	−225.11261	−225.10875	−225.25430	−225.18078	−225.17417
E+SI+I'	−225.26255	−225.19670	−225.19204	−225.33931	−225.26644	−225.25903
E+SI+I	−225.28283	−225.21770	−225.21307	−225.35992	−225.28764	−225.28027
E(SP)	−224.76124	−224.75219	−224.74756	−224.85752	−224.83597	−224.82929
E+SI(SP)	−224.91625	−224.90790	−224.90199	−225.01252	−224.99168	−224.98372
E+SI+I'(SP)	−224.97673	−224.96700	−224.96017	−225.07301	−225.05078	−225.04189
E+SI+I(SP)	−224.99011	−224.98034	−224.97344	−225.08639	−225.06411	−225.05517
	valence electrons correlated					
E+SI	−224.92613	−224.93930	−224.94483	−224.98425	−224.99564	−225.00109
E+SI (SP)	−224.88688	−224.90372	−224.90913	−224.94804	−224.96203	−224.96714

Table S8: Vertical excitation energy ($E(^3B_2) - E(^1A_1)$ in eV) of O_3 in cc-pVXZ basis sets, obtained by SLG-based methods.

	core and valence electrons correlated					
	$\hat{H}_{\text{ext2}}$			$\hat{H}_{\text{ext1}}$		
	cc-pVTZ	cc-pVQZ	cc-pV5Z	cc-pVTZ	cc-pVQZ	cc-pV5Z
PT2	1.425	1.435	1.440	1.419	1.429	1.434
E	0.883	0.863	0.837	2.497	0.859	0.860
E+SI	1.011	0.947	0.950	0.962	0.947	0.949
E+SI+I'	1.302	1.324	1.326	1.327	1.326	1.329
E+SI+I	1.422	1.446	1.449	1.447	1.450	1.452
E(SP)	0.870	0.876	0.875	0.864	0.871	0.870
E+SI(SP)	0.912	0.922	0.924	0.906	0.917	0.919
E+SI+I'(SP)	1.098	1.105	1.105	1.091	1.100	1.100
E+SI+I(SP)	1.167	1.174	1.174	1.161	1.169	1.169
	valence electrons correlated					
E+SI	0.947	0.953	0.957	0.947	0.953	0.957
E+SI (SP)	0.917	0.922	0.923	0.914	0.919	0.920
